# Band-Gap Regression with Architecture-Optimized Message-Passing Neural Networks

**DOI:** 10.1021/acs.chemmater.4c01988

**Published:** 2025-02-12

**Authors:** Tim Bechtel, Daniel T. Speckhard, Jonathan Godwin, Claudia Draxl

**Affiliations:** † Humboldt-Universität zu Berlin, Zum Großen Windkanal 2, 12489 Berlin, Germany; ‡ Max Planck Institute for Solid State Research, Heisenbergstraße 1, 70569 Stuttgart, Germany; § Orbital Materials, Oak House, Tanshire Park, Shackleford Road, Elstead GU8 6LB, Surrey, U.K.

## Abstract

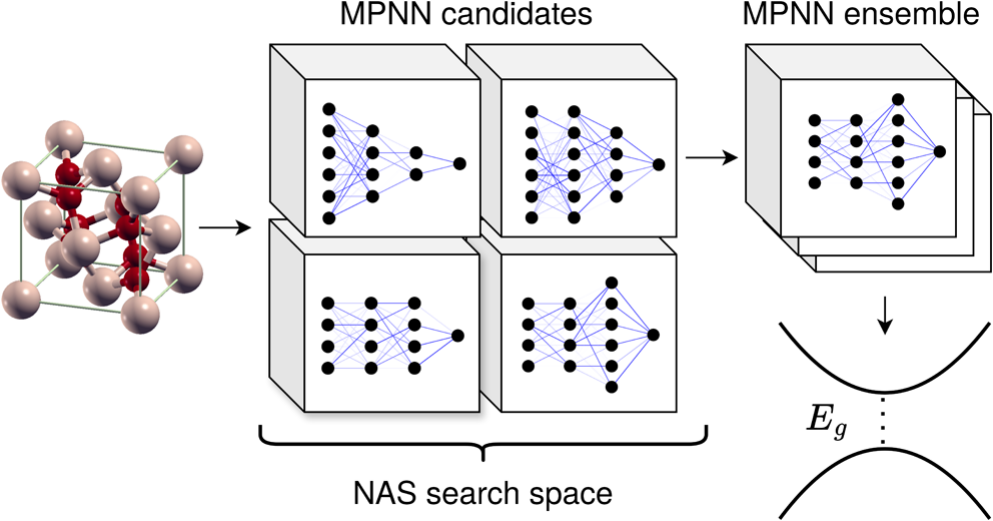

Graph-based neural networks and, specifically, message-passing neural networks (MPNNs) have shown great potential in predicting physical properties of solids. In this work, we train an MPNN to first classify materials through density functional theory data from the AFLOW database as being metallic or semiconducting/insulating. We then perform a neural-architecture search to explore the model architecture and hyperparameter space of MPNNs to predict the band gaps of the materials identified as nonmetals. The top-performing models from the search are pooled into an ensemble that significantly outperforms the best single model. Uncertainty quantification is evaluated with Monte Carlo dropout and ensembling, with the ensemble method proving superior. The domain of applicability of the ensemble model is analyzed with respect to the crystal systems, the inclusion of a Hubbard parameter in the density-functional-theory calculations, and the atomic species building up the materials.

## Introduction

The success of density functional theory (DFT) has allowed researchers to predict material properties outside of the laboratory. There are several materials databases, such as NOMAD (Novel Materials Discovery),^1^ the Materials Project,^2^ AFLOW (Automatic Flow of Materials Discovery),^3^ OQMD (The Open Quantum Materials Database),^4^ and others, collecting DFT data. NOMAD, for instance, contains over 150 million ground-state calculations. These databases have not only allowed researchers to avoid performing the same calculations again and again, thus saving computational resources, but have also enabled the repurposing of data.^5^ For instance, one can train statistical-learning models, e.g., regression techniques, compressed-sensing approaches,^6^ or neural networks (NNs),^7^ to predict DFT results with great accuracy.^8^ These databases contain formation energies and sometimes additional properties such as band gaps or elastic coefficients. Not every entry comes with a full set of calculated properties. This is due to these databases growing over many years with the addition of curated subsets intended for a specific purpose. AFLOW, for instance, contains roughly 3,500,000 calculated formation energies but an order of magnitude fewer band gaps. This provides an opportunity to compliment the published data sets by machine-learned properties using a fraction of the cost of DFT. These machine learning (ML) models need to be reliable for them to be useful.

In order to infer properties of solid materials, details of the crystal structure are essential. In graph neural networks (GNNs), the geometrical information, i.e., unit cell and atomic basis, can effectively be fed into ML models. GNNs learn a representation for each atomic element in the first layer of the network, the embedding. The embedding is then iteratively updated for each atom using information from neighboring nodes. GNNs have seen success in predicting energies of molecules,^9^ formation energies of materials,^10^ and interatomic potentials.^11^ While ML interatomic potentials have been developed with tremendous progress,^12−14^ less effort is going into training models on material properties other than energies and forces. Moreover, uncertainty estimates for these models are often missing. Overall, a common problem in machine learning is not only how to choose the hyperparameters, but also the network architecture. Choosing the right GNN architecture is no simple task, since there is a large variety of possible choices leading to different predictive performance.

In this work, we automate this task with a neural-architecture search (NAS)^15,16^ using a random-search algorithm that is agnostic with respect to the target property. We create a search space, based on two models, a message-passing neural network (MPNN) with edge updates,^17^ and the polarizable atom interaction neural network (PaiNN),^18^ covering a variety of hyperparameters and total parameter counts. In terms of training data, we focus on the AFLOW database, where both formation energies and band gaps are available for 62,102 structures. We first train an MPNN to classify materials as metal or nonmetal. We then run the NAS for band gap regression on the classified nonmetals. The NAS is also used to predict the formation energies. Exploring different ensembling methods and the optimal ensemble size, we find that pooling the best ten models from the NAS^19^ results in average predictions better than the best single model and existing models in the literature. The domain of applicability of the model is analyzed with respect to the atomic composition, the crystal structure, and the inclusion of a Hubbard parameter in the DFT calculation. Finally, as a means of providing the user with uncertainty estimates on individual predictions, we compare the performance of Monte Carlo dropout (MCD) estimates with ensemble uncertainty estimates. Finally, this workflow can be applied to make predictions on data where some material properties are missing. Here, we predict the band gaps on AFLOW data that are missing this property using the best performing model.

## Methods

### Graph Representation of Solids

**Figure 1:**
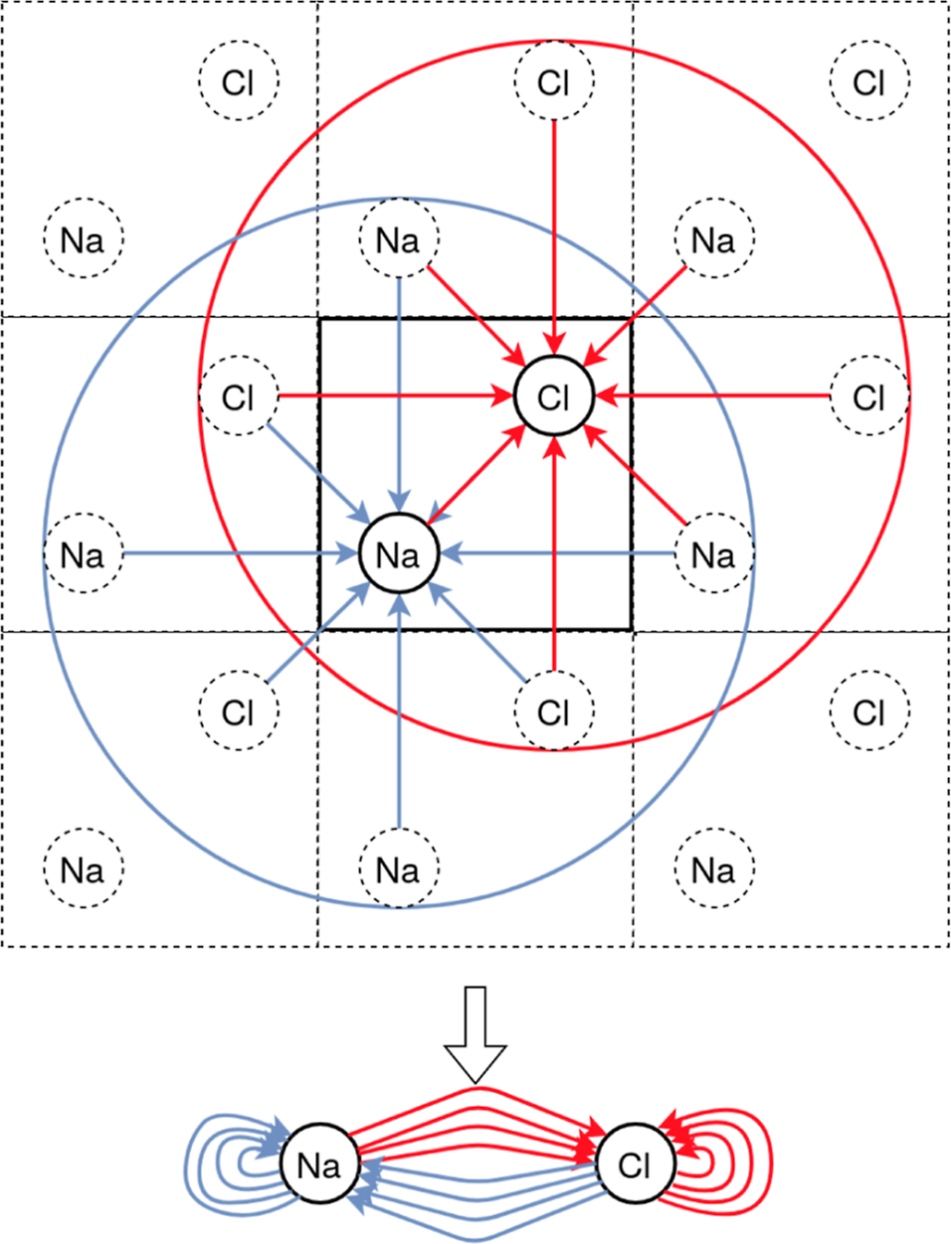
Construction of a graph representation (bottom) from the two-dimensional periodic unit cell of NaCl (top) using a fixed cutoff radius. The unit cell in the center is indicated by the bold square. Neighboring unit cells are also shown (dotted squares). The cutoff radii for Na and Cl, are shown with a red and blue circle, respectively, centered on their atomic positions.

A crystalline material is described by a periodically repeated unit cell. It is fully characterized by the lattice vectors, the involved atoms, and their positions in the unit cell. Graphs allow one to construct representations of physical systems that have translational and rotational invariance and are thus well suited for our purpose. In a graph representation, the local neighborhood of each atom can be defined by the distance to every other atom within a specified cutoff radius. When we apply this to a crystalline material, the cutoff radius may extend beyond its unit cell. An example of this can be seen in Figure 1 for the graph construction of two-dimensional NaCl. As all atoms within the unit cell, i.e., one Na and one Cl atom, represent nodes, we end up with two nodes in our example (bottom panel). All atoms within the respective circle are then connected to this respective atom via edges. Here, the Na node is connected with edges to four Cl atoms and four Na atoms in neighboring unit cells. The latter links are called self-edges that encapsulate the periodic boundary conditions of the crystal in the resulting graph, even though the graph representation itself is not explicitly periodic. Note that this graph construction uses directional edges where the edge originates from a node and ends on a node. Directional edges are used throughout this paper. Their use enables asymmetric graph representations, like a k-nearest neighbor graph, which is often favorable.

Once the nodes and edges of the graph are determined, we can define the adjacency matrix as
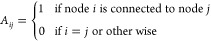
1

The neighborhood of node *i* is then formally defined as
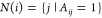
2

It is not possible to construct the adjacency matrix for periodic systems, as there can be multiple edges for a pair of atoms. Such a representation is not considered a simple graph (which has at most one edge per pair of nodes); rather, it is considered a multigraph (see Figure 1). However, we can still define the neighborhood of a node *i* by the set of other nodes to which it is connected.

Apart from using a constant cutoff to define the atomic neighborhood, the edges can also be constructed by considering a fixed number (*k*) of nearest neighbors (termed KNN algorithm). When the cutoff radius is the same for all atoms in the unit cell, the resulting graph is symmetrical. However, in the KNN algorithm, the constructed graph is not necessarily symmetrical but each node in the graph has the same number of neighbors. The KNN approach has several benefits. The cutoff radius can, in principle, produce isolated nodes, i.e., nodes without neighbors. As a reference to our studies, we refer to an architecture^17^ that was optimized to fit formation energies of structures present in the Materials Project database and OQMD, with the calculation of both being performed with the same DFT code and functional. In that work, it has been shown that a KNN cutoff with *k* = 24 neighbors produces the lowest mean absolute error (MAE) for an MPNN with edge updates when predicting formation energies on a OQMD materials data set, but the improvement over *k* = 12 neighbors is only marginal. In our experiments, training is sped up by about 20% using the lower number of neighbors, while not affecting the model performance significantly. Thus, in our search for an optimal message-passing architecture, we adopt *k* = 12, since—as we will show further below—this helps to reduce our already very large search space.

### Message-Passing Algorithm

MPNNs and, more generally, GNNs work by iteratively updating hidden graph states. They contain information concerning the atoms in the material and their interactions. In this work, we use hidden node and edge states which we refer to as node and edge vectors, respectively. Each hidden vector has its own update equation, which are the same as used in the reference implementation. After a fixed number of updates (*L*), the node vectors are fed into a readout function that predicts the target property.

#### Node and Edge Embeddings

The raw node and edge features are first transformed into representations that facilitate the graph network to learn from the input data. The atomic number, *Z*_*i*_, of each node *i* is one-hot encoded (OHE) into a vector. Its dimension is the number of different atomic species in the data set. For instance, in a data set containing 74 different elements of the Periodic Table of Elements (PTE), each atom is assigned a 74-dimensional binary vector, where only a single bit is nonzero (e.g., hydrogen is represented with [1, 0, 0, ..., 0] and helium with [0, 1, 0, ..., 0]). The element type, represented as an OHE vector is then transformed into a vector with latent size *C* by multiplication with a trainable weight matrix *W*_0_
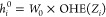
3With *W*_*i*_, we denote the weight matrices, whose elements are optimized during the training process.

Each edge in our graph is represented by a hidden edge-vector state. The initial state (embedding) is computed by feeding the pairwise atomic distance, *d*_*ij*_, between two nodes, *i* and *j*, into a basis-function expansion. As *d*_*ij*_ is always translationally and rotationally invariant, the graph reflects this desired property. In this work, we use Gaussian basis functions
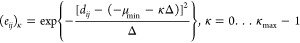
4The parameters μ_min_ (offset of the basis functions), Δ (width of the basis functions), and κ are chosen to span the range of input features. In the reference implementation, μ_min_ is set to 0 Å, Δ to 0.1 Å, and κ_max_ to 150. We use these values in this work as well. This dimensional expansion of the scalar distance into a vector of size κ_max_ might seem strange but is analogous to OHE in that the model is then able to decorrelate input and output more easily with the transformed, now higher-dimensional input.

#### Node/edge Update Functions

The nodes and edges are updated at each message passing (MP) step *l*. First, the edge update is performed, then the node update is applied using the updated edges. For each edge, *M*_*ij*_ is defined as an edge-wise message that connects node *i* to node *j* (node *j* is sending, and node *i* is receiving the message)
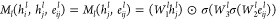
5Here, the symbol ⊙ denotes element-wise multiplication, and σ an arbitrary, nonlinear activation function. The element-wise multiplication can be seen as a continuous filter, where the edge feature attenuates the node feature, after both have been transformed by feed-forward layers.

Edge-wise messages are aggregated into node-wise messages by either taking the sum of neighboring features as in
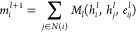
6or any permutation-invariant aggregation function (e.g., mean, minimum, maximum, etc.).^20^ The edge-update function consists of concatenating the sending and receiving nodes with the edge feature *e*_*ij*_. This concatenation is then passed into a two-layer NN with two shifted soft-plus activation functions
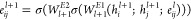
7

Nodes are then updated according to
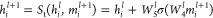
8by using the aggregated messages *m*_*i*_^*l*+1^ and the original node features *h*_*i*_. The node-wise message is transformed in a two-layer NN with an activation function and is added to the previous node feature *h*_*i*_^*l*^, to arrive at the updated node feature *h*_*i*_^*l*+1^. This addition has similarities to the residual connections used in ResNet architectures, which enable training of deeper NNs.^21^

#### Global Readout Function

After *L* MP steps, the procedure is stopped, and the node features are aggregated into a single scalar, transforming them by means of an NN with two layers and a hidden size of *C*/2. Subsequently, one sums over all nodes in the graph or takes the mean. This step is required since the aggregation should be invariant with respect to the permutation of nodes, as their ordering should not matter. Whether the sum or the average is taken over all nodes depends on the data set and the target property. Here, we show the equation for the summation
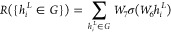
9

As the graphs can have variable sizes, the aggregation should also be able to handle varying numbers of nodes in the graph. Note, for the QM9 data set, where the target is the total internal energy *U*_0_, we use a sum in the readout function. For the data sets, where the formation energy per atom is targeted, we take the mean. For further discussion on readout aggregation methods, see ref (22).

Combining the pieces of the node/edge embedding, the node/edge update functions, and the readout function, we arrive at the complete algorithm for a message-passing edge-update neural network, abbreviated as MPEU:
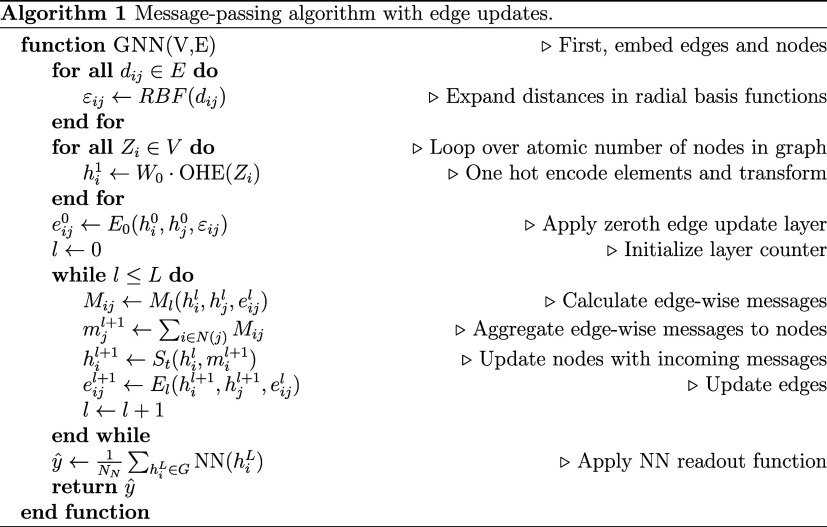


### Architecture Search

MPNNs as described above contain many architecture parameters. Not much work has been devoted to explore how they affect the model performance. In this work, we perform a neural architecture search (NAS) using a random search algorithm. We build our search space based on the MPNN model described in detail above, where the embedding and latent size (e.g., the node/edge vector dimension), the number of MP steps, the activation function, the number of layers in the MLP in the node/edge update functions, and the number of layers in the readout NN are varied. Other MPNN parameters, such as the initial learning rate, the learning-rate decay, the batch size, the dropout and the layer norm are also varied concurrently, assuming that the importance of these variables is related to the number of parameters in the model. We note that the number of trainable weights, i.e., parameters optimized during training, scales linearly with the number of MP steps, and quadratically with the latent size. The number of trainable parameters (weights) ranges from 500,000 up to 20,000,000, with the best models usually having around 1,000,000 weights. Neural architecture searches are often performed with a mix of explorative and exploitative algorithms such as Bayesian optimization or genetic algorithms.^23^ In this work, we opt to use a random search algorithm since we want to sample the large multidimensional space exploratively to gain a better understanding of the space. While Bayesian optimization and genetic algorithms sample the parameter space with a bias toward regions with well-performing models, random search samples the parameter space without bias, i.e., purely exploratively.

### Neural-Network Ensembles

Given that a large number of models is trained in the process of the NAS, it is a natural step to not only look at the best performing model, but also at the predictions of the other models. If a number of well-performing and diverse models make a prediction on a single input, it can be expected that the average prediction of the models in the ensemble outperform the individual models.^24,25^ There are three main reasons for this:^19^ (i) different models can achieve the same performance on the regression or classification task. An ensemble reduces the risk of choosing the poorly performing model when it is applied on the held-out test data. (ii) Due to the nonconvex nature of optimizing a neural network, it is expected that training results in a local minimum with respect to the trainable parameters rather than the global minimum. This leads to the possibility of different locally optimal parameters given the same training data, if the initialization of the models is different. Again, the model ensemble reduces the risk of choosing a poorly performing model that is stuck in a local minimum far from the global minimum. (iii) By averaging across different models, the space of possible solutions is expanded, leading to an increased learning capacity of the model.

The main prerequisite for an ensemble to improve prediction quality is that the models are diverse, i.e., are trained with different data and/or have different parameter values and or architectures, and that the different models are well performing on their own. For our ensemble, we select the ten best candidate architectures with respect to the validation data set from our NAS. We show that the breadth of architectures of the different candidate models together with the corresponding hyperparameters results in an ensemble that is less prone to overfitting and performs better than the top NAS models individually.

### Uncertainty Estimates

Reliable uncertainty estimates are important when deploying a machine-learning model in a real application.^26^ They provide information on the model’s domain of applicability so that the user can understand whether to trust an inference.^27^ Ensembling the top ten models from our NAS gives us a method to obtain an uncertainty estimate by looking at the predictions from all ten models in the ensemble and calculating the standard deviation. We compare this method with another popular method, Monte Carlo dropout (MCD).^28^ Dropout means that nodes in the network are turned off/on probabilistically. In the case of MCD, the dropout is also used for model inferences (i.e., nodes are turned off randomly for each prediction the model makes) and not just for training the model. This enables stochastic predictions from a virtual ensemble. Ideally, aggregating these predictions gives an uncertainty estimate in the same way as a Gaussian process would. We employ dropout on all NN layers in the model (readout function, edge-update function, etc.). Dropout also helps to prevent overfitting during training for regularization purposes.^29^ The dropout is kept on for inferences; ten predictions are made for each input, of which the mean and standard deviation are reported.

### Band-Gap Classification and Regression

The Kohn–Sham band gaps obtained in density functional theory typically severely underestimate the corresponding quasi-particle gaps of the respective structures. This systematic error can be partly remedied by the use of hybrid functionals,^30^ but an expansive database has yet to be created using this method. It is expected, however, that a model that is fitted on biased data, carries the same bias during inference on unseen data. This should be kept in mind when discussing the use of GNNs trained on DFT data in high-throughput searches, e.g., for large-band gap materials. To make sure that we use as consistent a data set as possible, the data used in this work were filtered to only contain DFT calculations performed with the PBE^31^ functional. For more details on the data, see below.

**Figure 2:**
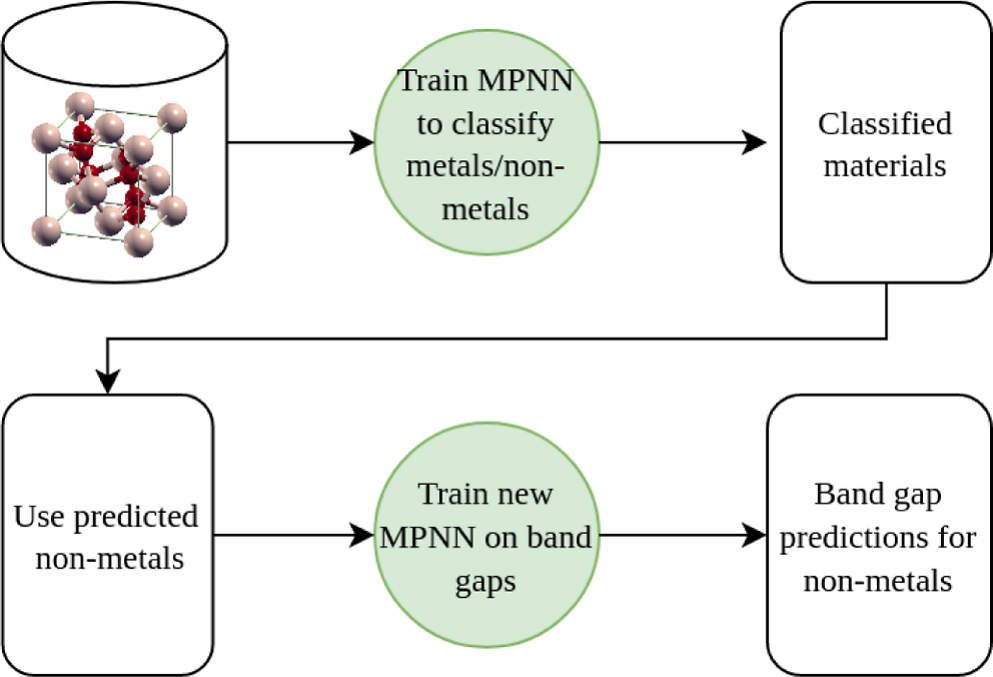
Workflow of band gap classification in terms of metals/nonmetals using an MPNN, followed by the prediction of band gaps using another MPNN.

Following the literature on predicting band gaps on AFLOW data using the PLMF (Property-Labeled Materials Fragments),^32^ we train two separate models. The first one classifies materials as nonmetals and metals (having a zero DFT band gap), using a binary cross-entropy loss. The second model is fitted to predict the band gaps of the materials classified as nonmetals. This workflow is illustrated in Figure 2. Both models—classification MPNN and regression MPNN—have a similar architecture. Since we find very high accuracy on the classification task without tuning the hyperparameters, we only perform a NAS on the band gap regression task. The hyperparameters of the classifier match the ones of the reference model, which was optimized to predict formation energies on the Materials Project database. Unlike the reference model, the readout function of the classifier model (see eq 9) has two (instead of one) output values in order to be compatible with the binary-cross-entropy loss used to train the classifier.

### Data Set

For band gap prediction and classification, we use all materials in AFLOW that have a band gap. The AFLOW data are obtained with the DFT code VASP.^33^ As mentioned above, we only use those calculated with the PBE functional, and duplicates have been removed from the data set. To simplify our analysis, we use the same data set for the prediction of formation energies. That means that there are some materials for which a formation energy has been calculated but no band structure, and they are therefore excluded from the formation-energy regression.

Outliers with a formation energy of less than −10 eV/atom (i.e., two materials, S and SiO_2_, both space group 70) or higher than 70 eV/atom (a single material, BrIPb, space group 59) were removed. These outliers have formation energies more than five σ away from the mean in the data set. In this data set, we have 46,090 metals and 16,012 nonmetals. The data set is therefore biased toward metals.

### PLMF and ElemNet Models

To evaluate and compare the performance of the models from our NAS, we include several models from the literature. In the PLMF model, the lattice structure was decomposed into fragments, and a ML model was trained on these fragments. To the best of our knowledge, the PLMF model is the only model in the literature that has been used to classify band structures and regress band gaps for the AFLOW data set. One caveat to the comparison with this work is that neither the training/test splits, nor the code for the method employed in their work were shared in the original paper. We compare our results to the metrics reported for PLMF, however, we note that their data set is from an earlier snapshot of AFLOW. Since the publishing of that article, the AFLOW database has grown and some data points have been recomputed (e.g., with a newer version of the VASP code). More specific, we used the online API^34^ to get results from their model trained with the earlier AFLOW data set snapshot, evaluated on the current AFLOW test data set. Therefore, not having access to the training/test splits used by the PLMF model, some of the test data might have already been seen by the trained model.

We also compare our results with the deep neural network ElemNet,^35^ a model that does not use any structural information, but is given only the chemical formula (stoichiometry). This model was trained and evaluated using OQMD^36^ data and demonstrated good performance for formation energies. The problem of having two or more different crystal structures with the same chemical formula was avoided by including only the structures with the lowest formation enthalpy. In this work, we do not follow this strategy in order to compare the ElemNet architecture fairly with our other models. We retrain the model on AFLOW data but keep the model architecture from the original publication. The initial learning rate for the Adam optimizer was varied between 1 × 10^–3^ and 1 × 10^–5^, and the value that yielded the best performance on the validation data set was chosen. We also include a version of the ElemNet model architecture, which we call ElemNet-Phys, that takes in physical attributes as input. They are described in the original publication, but are only used with a Random-Forest model.

## Software Implementation

For our computational framework, the JAX ecosystem is used because of its frequent use in recent state-of-the-art research.^37,38^ Features like automatic differentiation and just-in-time compilation, and active development that foster new scientific discoveries are especially appealing. In conjunction with JAX, we use the Haiku library for trainable NN layers^39^ and Optax for optimization routines which is itself built upon JAX.^40^ Finally, to encode our MPNN architecture and update equations we use Jraph which provides a functional API to apply transformations to arbitrary graphs.^41^ These four libraries form a cohesive framework for the whole process of training a graph-based machine-learning model, apart from the database interface that we implemented.

As a local database for atomic structures, we employ the Atomic-Simulation-Environment database (ASE-DB).^42^ The conversion of atomic structures to graphs is done only once for each database using a maximum neighborhood of k-nearest neighbors, therefore the time-consuming graph generation does not have to be repeated for each hyper-parameter experiment.

The data are divided into training, validation, and test data in an 80:10:10 split. Training and validation data are used for cross-validation and early stopping, and the model is finally evaluated on the unseen test data to asses its performance on samples it has not yet encountered. Early stopping is implemented as described in,^17^ by checking if the validation loss has decreased compared to the loss 1 million steps before. The model is trained with dynamic batches of a maximum of *N*_batch_ graphs (including a padding graph)^43^ using the Adam optimizer provided by Optax.^44^ Batches are sampled without replacement from the training data set and reshuffled in every epoch. Dynamic batches are created by calculating the average number of nodes and edges for *N*_batch_ – 1 graphs and rounding this result up, in this case to the next multiple of 64. This value (power of 2) is motivated by the processor architecture that is used, in that GPUs use banked memory and specific optimized kernels that work best with data sizes of 2^*N*^. Then, during the training loop, graphs are sampled without replacement from the training data set, until the maximum number of nodes or edges is reached, or *N*_batch_ – 1 graphs are retrieved. The rest of the budget is then used for padding, and the result is a static number of nodes, edges, and graphs. This only needs to be just-in-time compiled once and therefore greatly increases the speed of each graph network evaluation.

The implementation is validated by training a model on formation energies from the Materials Project, specifically the MP-crystals-2018.6.1 snapshot provided in.^45^ With this training data, we obtain similar error metrics to ref (17) despite using a different training/test split (no information on the split used was provided in ref (17)). The code to perform the NAS, along with data retrieval, data processing, model training, and prediction analysis used in this manuscript is available on GitHub.^46^

In order to compare the MPEU model with a more recent model from the literature, we train a PaiNN model,^18^ which is a GNN that uses both invariant and equivariant representations of the atomistic structure and includes directional information in addition to the distance information in the message passing. We use the implementation in ref (47) and integrate it into our own code, with some changes in the training procedure, compared to the training in the original publication, i.e., we use an exponentially decaying learning rate, we do not smoothen the validation curve for model selection, and we use the ADAM optimizer. From our experiments, these changes do not seem to affect the model performance significantly.

## Results

We first train a model to classify materials as metals or nonmetals, minimizing the binary cross-entropy. We evaluate the receiver operating characteristic (ROC) alongside the total accuracy of the model, since these two metrics are easier to interpret than the binary cross-entropy. The area under the curve (AUROC) of the receiver operator characteristic presents a balanced scalar metric of the classifier’s performance. Recall, that an AUROC of one indicates a perfect fit, whereas an AUROC of 0.5 signals the model is making random predictions. The reference MPEU model performs quite well for the classification task with an accuracy of 0.98 and an AUROC over 0.99 as shown in Table 1. This is remarkable since the reference MPEU model was not optimized for classification. Both the AUROC and accuracy are higher than the corresponding values for the PLMF model from the literature that are included for comparison. The high AUROC value indicates a very balanced classification performance across metals and nonmetals, despite the data set being biased toward metals. As a result of the satisfactory performance of the MPEU reference model, we decided not to perform further optimizations on the model with a NAS.

**Table 1:** Summary of Cross-Validated Models on Formation Energies (*E*_f_) and Band Gaps (*E*_g_) by Our Best Performing MPEU and PaiNN Models from the NAS^a^

property	model	RMSE	MAE	MdAE
*E*_g_ [meV]	MPEU: ensemble	379	168	26.2
	MPEU: best in NAS	469	205	35.0
	MPEU: baseline^17^	399	180	32.9
	PaiNN: ensemble	**368**	**158**	**22.9**
	PaiNN: best in NAS	399	177	34.7
	PaiNN: baseline^18^	381	171	29.2
	SchNet^48^	489	235	68.4
	PLMF (new data)*	1327	618	151
	PLMF (reported)	510	350	
	ElemNet	2492	636	1673
	ElemNet-Phys	816	515	303
	ElemRF	794	469	1581
*E*_f_ [meV/atom]	MPEU: ensemble	56.3	**15.0**	**6.29**
	MPEU: best in NAS	65.4	21.0	10.7
	MPEU: baseline	57.5	17.9	8.32
	PaiNN: ensemble	55.0	17.1	6.45
	PaiNN: best in NAS	61.0	21.6	9.83
	PaiNN: baseline	60.6	22.1	10.3
	SchNet	68.0	29.3	17.2
	ElemNet	638	834	1769
	ElemNet-Phys	214	135	68.6
		accuracy		AUROC
classification	MPEU: baseline	**0.98**		>**0.99**
	PLMF (new data)*	0.97		
	PLMF (reported)	0.93		0.98

a For comparison, PLMF model was evaluated on the new AFLOW data, using the web API, and results from the original publication, using an old snapshot of the AFLOW database are included (marked with *). Results of the classification into metals/non-metals are shown at the bottom. (The publication on PLMF did not provide MdAE value, and the PLMF API does not return probabilities, thus there is no AUROC value).

**Figure 3:**
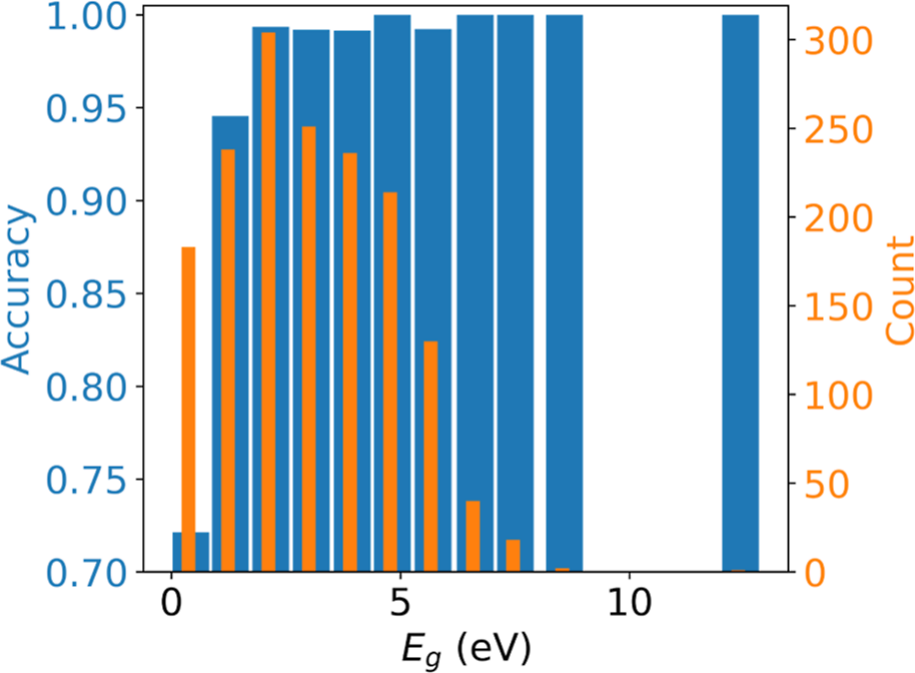
Histogram of band gaps (orange) and accuracy of the classification in metals/nonmetals, depending on band gap range, evaluated on the test data. The *x*-axis shows the DFT values. Not shown in the figure is the accuracy for DFT calculated metals, which is over 99%.

The classification accuracy of the model clearly depends on the band gap of the material, as shown in Figure 3. Not surprisingly, the model is less accurate near the classification boundary (DFT band gaps slightly above zero). The model does well for large band gap materials despite there being fewer data points in the training set.

**Figure 4:**
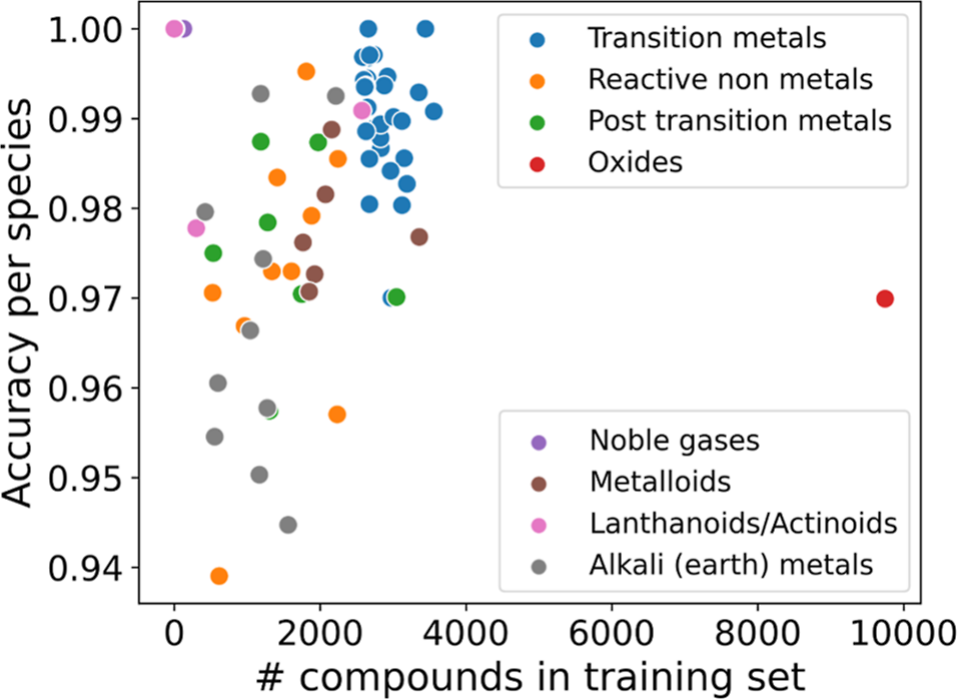
Accuracy of the classification between metals/nonmetals for different material classes evaluated on the test split.

In Figure 4, the performance of the classifier in terms of accuracy is analyzed depending on how often each type of material appears in the training split. As expected, we see a general trend that the more often that a category appears in the data set, the higher the classification accuracy for that category. For instance, transition metals appear frequently in the training split and are generally classified correctly with more than 98% of the time. In contrast, the fewer alkali metals are classified with accuracies ranging from 94% to 99%. Oxides, however, are outliers in this trend. Despite being best represented in the training data set with over ten thousand materials, they are classified with an accuracy of 97%, lower than the mean accuracy over the entire data set (98%). One reason for this could be the fact that for many of the transition metal oxides, a Hubbard-*U* correction has been applied in the production of the DFT data. We will come back to this point further below.

**Figure 5:**
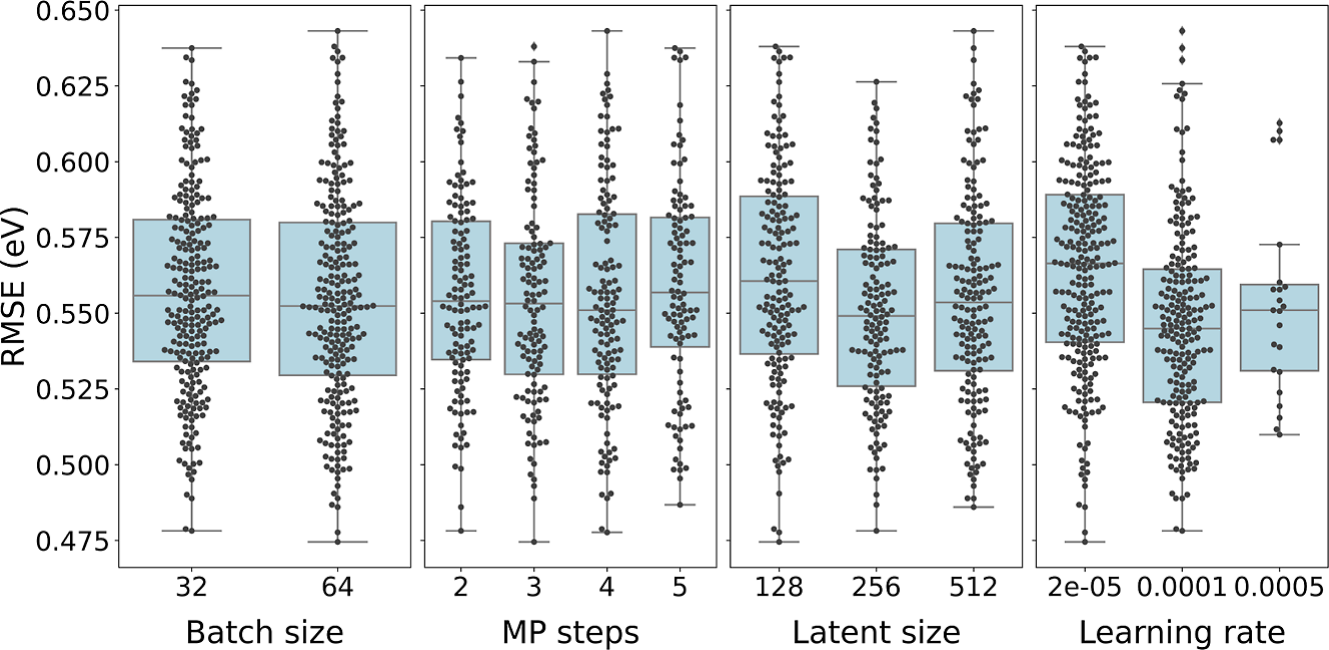
MPEU-based neural architecture search results for the band gap, using the nonmetals as predicted by the classifier (trained on materials from the AFLOW database). The RMSE on the validation split are shown for model-architecture parameters and settings in our search space. The effect of several additional parameters is shown in the Supporting Information.

After classification, we predict the band gaps of those materials that have been classified as nonmetals. We visualize in Figure 5 the results of the neural-architecture search for band gap regression on the validation split. In order to compare different base models, the NAS is performed for the MPEU model as well as the PaiNN model. Validation results for the latter as well as additional hyperparameters for the MPEU are provided in the Supporting Information. We first discuss the hyperparameters of the MPEU model for which the NAS was performed more extensively. The results indicate that, in general, the larger batch size of 64 is only slightly better than 32. Three message-passing steps give the lowest mean RMSE. A latent size of 256 is favored and a learning rate of 1 × 10^–4^ is significantly preferred. Although, for instance, a latent size of 256 and a learning rate of 1 × 10^–5^ are preferred on average, the best NAS model has a latent size of 128 and a learning rate of 2 × 10^–5^ which demonstrates the correlation between the architecture and hyperparameters. We see that increasing the latent size and using a smaller learning rate, which should increase the model’s learning capacity, does not result in a lower RMSE. This may hint at a rather small amount of training data for the problem. In total, 500 random models were trained; for 459, the loss converged and training was stopped early; for 41, the optimization was aborted due to an unstable loss value (as often observed by us when a higher learning rate is paired with no layer normalization). For the PaiNN architecture, we trained only 100 models since this led to satisfactory results. From these models, 93 were stopped early due to convergence of the loss function, three runs were aborted, and four runs reached the maximum number of gradient steps of ten million. The NAS model that performed best on the validation data set in terms of RMSE was selected as the model used for testing, and the best ten models are used to create ensembles.

The regression metrics, RMSE, MAE, and median absolute error (MdAE), are collected in Table 1. The MdAE is an error metric that is unaffected by outliers, as it gives a midpoint where the same number of absolute errors lie above and below. The low MdAE value across models shows that both the RMSE and MAE are affected by outliers with a high absolute error. We observe that the best NAS architecture is overall similar to the reference model, however, the optimal learning rate at 2 × 10^–5^ is lower, and the NAS model uses a dropout rate of 0.05 while the reference does not use any. The best NAS model actually performs worse than the reference model on which the NAS space is created. A table comparing the validation results and the test results is shown in the Supporting Information.

**Figure 6:**
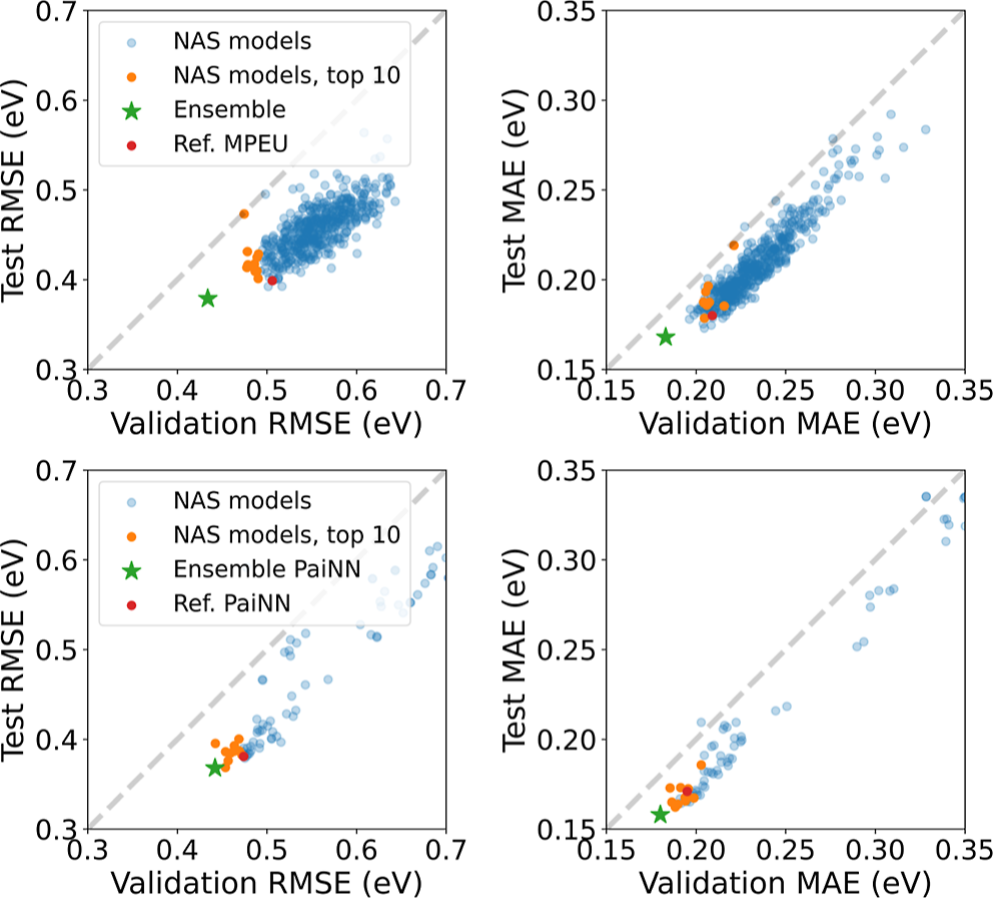
Comparison between validation split and test split RMSE (left panels) and MAE (right panels) on the band gap target from the NAS on the MPEU (top panels) and PaiNN (bottom panels) search spaces. The reference model and the ensemble of the best NAS candidates are also included. The dashed gray lines indicate where the data points should lie if the validation and test sets were infinitely large, and had the same distribution.

The validation and test RMSE are very similar for the best NAS model (468 and 469 meV, respectively). We conclude that it is not overfitting the validation data set although our NAS selects the best model based on the validation RMSE. In contrast, for the reference model, the validation RMSE is much higher than the test RMSE (505 and 399 meV, respectively). This suggests that the reference model’s superior test performance may depend on the split. Our ensemble model that combines the top ten NAS models, outperforms the reference model significantly in terms of MAE, RMSE, and MdAE. Note that in order to evaluate all band gap regression models on equal footing, we use the same MPEU band gap classifier, so that each model uses the same training, validation, and test data set. As we see a significant difference in performance of the models depending on which split they are applied to, we plot the performance of the models on the test split, depending on the validation score, in terms of RMSE and MAE in Figure 6. Both plots show that the models generally perform slightly better on the validation data than on the test data. We observe that the test and validation scores are more correlated when evaluated using the MAE, while the RMSE scores are more scattered, meaning that there is less correlation between test and validation RMSE. This is likely due to outliers, to which the RMSE is more sensitive. The ensemble model outperforms all NAS models on both splits and both metrics. The reference architecture MPEU performs well on the test data, in terms of RMSE, while it is not within the top ten models when evaluated on the validation data and is one of the models with the largest difference in validation and test RMSE. These observations lead us to conclude that the reference model is rather fortunate to achieve such a low test RMSE. Overall, ensembling helps to achieve significantly better validation and test RMSE than any of the candidate models.

**Figure 7:**
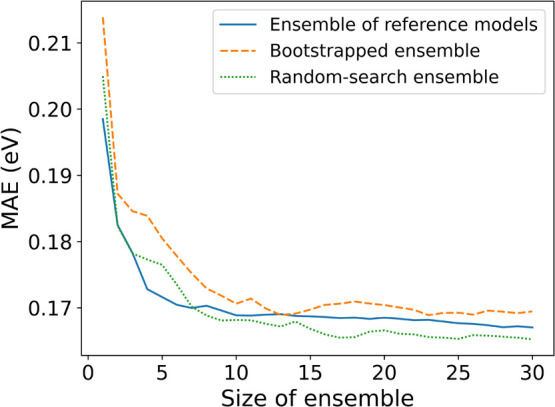
Ensemble performance (MPEU architectures) on the test split for the band gap regression task. The bootstrapped ensemble is a collection of multiple copies of the reference architecture trained separately on randomized training/validation splits, and with a random weight initialization. The reference ensemble is the same reference MPEU architecture with a different weight initialization but with the same training/validation split. The random-search ensemble refers to the *N* best models ensembled from the NAS.

We also investigate how the number of ensemble models affects the test performance, by ensembling the top *N* MPEU models. No significant improvement in test MAE is observed when ensembling more than ten models (see Figure 7). The test error of the NAS ensemble is compared with that of an ensemble built from reference MPEU models, meaning that the architecture is the same in all models in the ensemble. For this, we evaluate two methods. The first uses *N* models trained on the same training/validation split with a different random initialization. The second is a bootstrapped ensemble,^49^ where the validation split is sampled (with replacements) from the training split separately for each individual model, while the test split remains the same in all experiments. We find that the reference ensemble performs better than the bootstrap ensemble, but both are worse than the random search NAS ensemble when the size of the ensemble is larger than seven. The fact that the NAS ensemble does not perform well until we increase the ensemble size suggests that when we pick the best models for the ensemble based on their validation performance, we are likely choosing models that overfit the validation data set. Ensembling appears to help avoid overfitting the validation data set and improve the test results.

**Figure 8:**
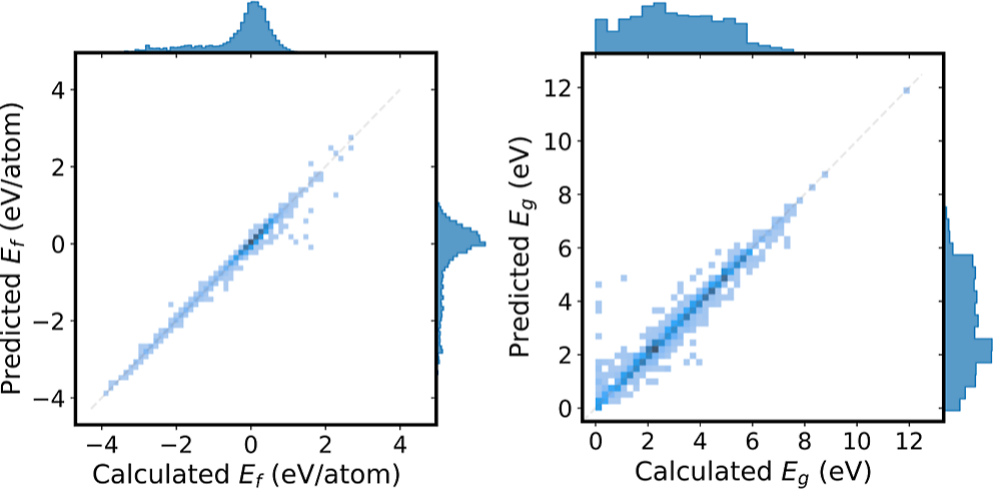
Regression results using the ensemble PaiNN model on AFLOW data for formation energies (left) and band gaps of predicted nonmetals (right). Marginal histograms showing the distribution of predicted and calculated values are shown above the horizontal axes and to the right of the vertical axes. MAE values (as listed in Table 1) correspond to the average absolute distance to the diagonal.

Analyzing the performance of the ensemble model further, we see that the largest RMSE occurs on data that our classification model incorrectly predicted as nonmetals. This is seen in Figure 8. All MPEU and PaiNN models significantly outperform all other models (PLMF, ElemNet, and ElemNet-Phys), indicating the superiority of graph-based models for this task and data set. The crystal structure also plays an important role in the quality of the MPEU model for predicting band gaps, as shown in Figure 10. In general, we see a better prediction for lattice types with more materials in the training set. For instance, cubic systems are quite common and have the lowest median error while triclinic structures are the most rare and have a poor median error. That said, there is a similar number of hexagonal training structures compared to triclinic structures (792 vs 603) but we observe a much lower median error for the former. The dependence of the model performance on the lattice type, on the other hand, indicates that the model is learning from the input crystal structure, which is desired.

**Figure 9:**
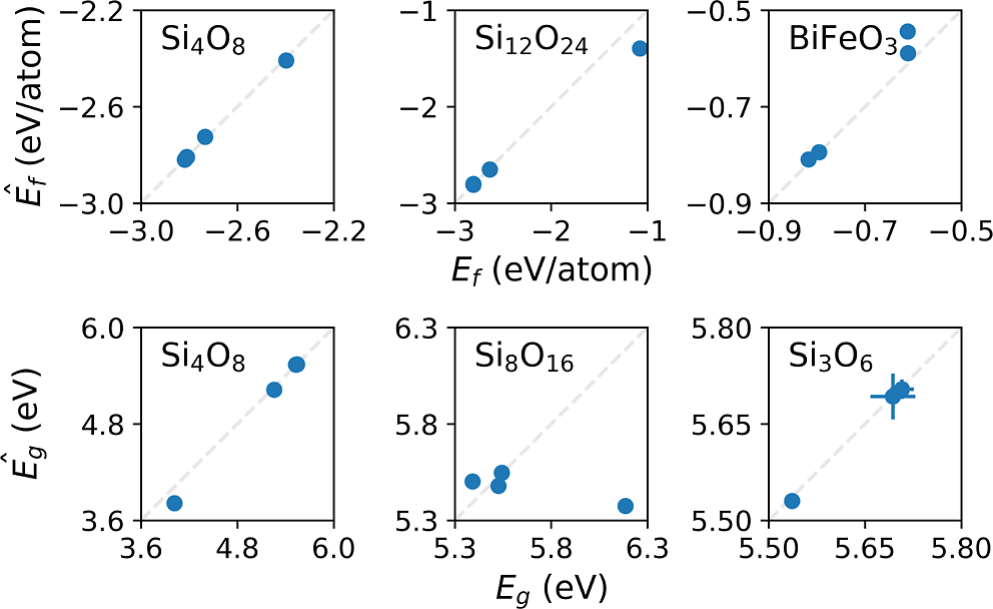
Regression performance when applying the best formation-energy (top row) and band gap models (bottom row) to the most common polymorphs and chemical formulas in the data set. Each data point corresponds to a unique space group. Ideal predictions are represented by the dashed gray lines. There are some AFLOW data points, where the chemical formula and space group are identical but computational details differ. For these data points the predictions (*y*-axes) and DFT-targets (*y*-axes) are averaged for visual clarity, and error bars show the standard deviation. Note that the band gap data set is a subset, only containing predicted semiconductors.

**Figure 10:**
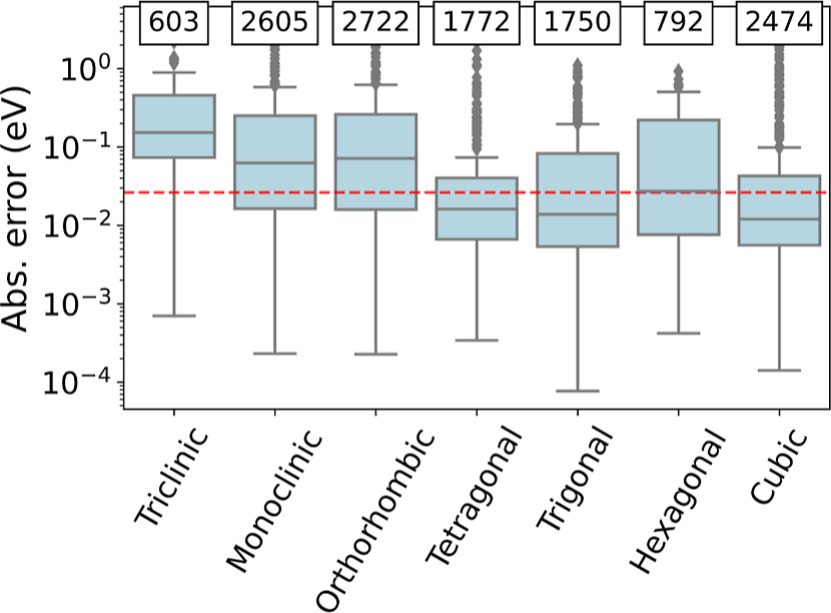
Distribution of absolute errors in band gaps for different crystal systems using the MPEU NAS ensemble model. The number of training materials for each crystal system is displayed at the top. The dashed red line shows the MdAE. Points above the 95% quantile are shown to better understand the distribution.

We also train our models to predict formation energies, performing a NAS on this task. The results are shown in Table 1; the effect of several NAS parameters is shown in the Supporting Information. They exhibit similarities to those of the band gap regression. The ensemble NAS models perform the best, but the reference model outperforms the individual top-ranked NAS model for both NAS search spaces. As the reference model^17^ was trained on formation energies of the Materials Project, it is no surprise that its architecture transfers very well to another data set based on the same code. The two databases show, however, significant differences in computational details, such as convergence criteria for geometry optimization, or the use of DFT + *U*, and alike, as analyzed in detail in ref (50).

An important aspect when predicting formation energies is the identification of the lowest-energy structure for a specific chemical composition. For this analysis, we consider structures in the data set that have the same chemical formula but different space groups. Note that for a few chemical formulas in the data set, such as Si_3_O_6_, multiple structures with the same space group and formula exist in the data set. This is due to a variety of computational parameters being used in the AFLOW workflow that yield very similar but slightly different structures (e.g., a difference in lattice parameters of 0.01 Å). In these cases, we take the average ML prediction and DFT target. This results in 39 polymorphs in the test set. The best individual PaiNN NAS model correctly identifies 36 out of 39 (92%) energy minima. The PaiNN ensemble model does slightly worse and predicts 33 (85%) correctly, despite the fact that the ensemble has better regression metrics. This indicates that a lower MAE of the regression does not necessitate a better identification of lowest-energy structures.

We can visualize the trends in the formation energies and band gaps, obtained by the best model (PaiNN ensemble) compared to the DFT targets, for the most common polymorphs in the two data sets in Figure 9. For the chemical formulas where multiple structures exist in the same space group, the average DFT target and ML prediction are shown for visual clarity. In general, we see good agreement between the ML models and the computed values.

**Figure 11:**
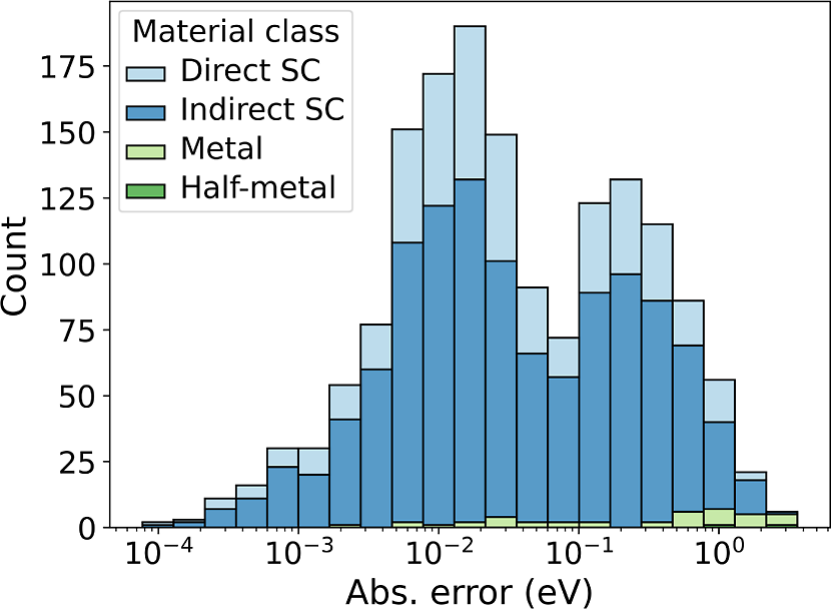
Distribution of absolute errors for different material classes, when predicting band gaps using the MPEU NAS ensemble model. Materials are grouped into direct semiconductors (direct SC), indirect semiconductors (indirect SC), metals, and half-metals. These classes refer to the true classes of the data, not the classifier’s prediction.

Uncertainty quantification is also provided by the MPEU/PaiNN models and ensembles. For the individual NAS models with a nonzero dropout rate, we perform MCD, while for the ensemble model, we get the variance in the predictions of the individual models. As a nonzero dropout rate was not tested for the PaiNN model, we compare MCD and ensemble uncertainty using the MPEU results in what follows. For band gap regression, we find that the uncertainty of the ensemble has a correlation of 0.63 with the absolute error. In general, the uncertainty underestimates the true error, which is a known problem with uncertainly quantification in neural networks.^51^ The MCD method of the best NAS model performs much worse with a correlation of 0.38. To better understand the problem of uncertainty quantification, we look in Figure 11 at the distribution of absolute errors of the ensemble model. In the test data set, 60% of the absolute errors are below 50 meV. At this level, we start to approach the numerical precision of DFT-PBE band gaps, and much of the uncertainty we are trying to predict may be irreducible, i.e., aleatoric, or just noise. Similarly, for the formation energy, most of the absolute errors are below 10 meV and thus also in the range of the numerical precision of the DFT data. It may therefore not be surprising that the formation-energy models have an even worse correlation with the uncertainty of 0.53 and 0.24 for the ensemble and best NAS model, respectively.

**Figure 12:**
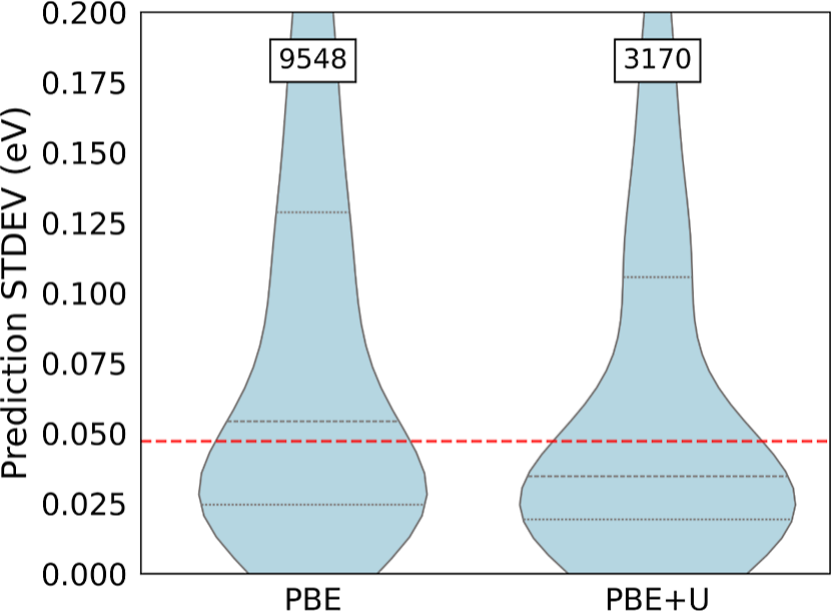
Violin plots of the standard deviations obtained by ensembling PaiNN NAS models when predicting band gaps of AFLOW data computed by either PBE or PBE + *U*. The red horizontal line shows the median standard deviations of the predictions over the whole test split, dashed lines show quartiles. The numbers of training examples are shown at the top.

For band gap prediction, the ensemble uncertainty is well correlated to whether the simulation was performed with a Hubbard *U* correction or not. This correction is applied for strongly correlated materials, where the PBE functional is known to perform poorly. In the AFLOW database, PBE + *U* is used for systems with d and *f* bands where electron localization occurs via the splitting of the energy levels of these orbitals.^52^ The impact of this method on the learning is seen in Figure 12 where the violin plots show the distribution of the model’s uncertainty estimate with respect to the *U* correction. The width of each curve corresponds with the empirical probability (i.e., relative frequency) of the magnitude of the inference uncertainty. When the ensemble uncertainty method is employed for the prediction of band gaps, the median standard deviation (center of the boxplot) is lower for materials with the Hubbard-*U* correction (see the Supporting Information for the plot). This higher confidence of the model for band gaps performed with the Hubbard *U* correction in comparison to the results for formation energies with the correction, indicates that the latter data are less consistent, as described in the AFLOW database.^53,54^ These observations are supported by the fact that also the absolute errors depend on the regression task. Formation energies (band gaps) are worse (better) predicted by the ensemble model for materials where the correction is applied.

The ElemNet-Phys model is superior to the ElemNet model in predicting band gaps and formation energies, which shows that the latter struggles when learning only from the atomic composition alone. This does not appear to be the case for the MPEU models, which are not fed physical attributes but given interatomic distances as features. The superiority of the MPEU and SchNet models over the two ElemNet models is not surprising, since the interatomic distances allow different crystals with the same chemical formula to be distinguished.

In order to understand why the MPEU models work so well for both the band gap and formation-energy regressions, we remove the edge updates in our algorithm, making it equivalent to the SchNet model. For our implementation of SchNet we use a reduced latent size of 64. This reduction in model size is needed in our case in order to converge the validation loss during training. We observe that SchNet performs superior to both ElemNet variants but falls short with respect to the MPEU models, supporting the hypothesis that edge updates increase the learning capacity of message-passing models significantly. The results are included in Table 1.

**Figure 13:**
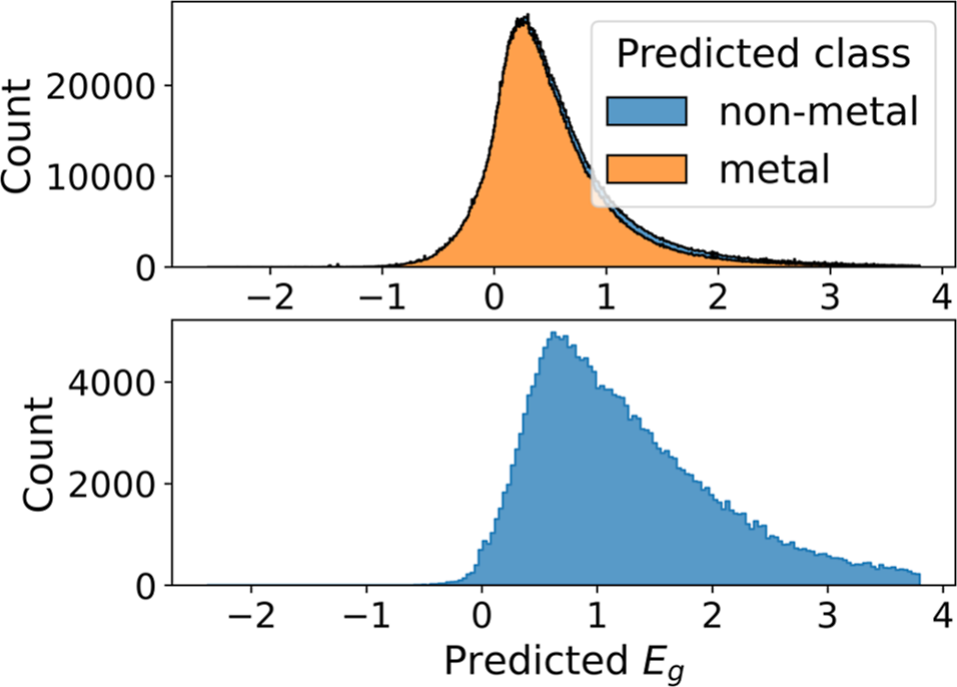
Histogram of predicted band gaps when the PaiNN ensemble is applied to the full set of AFLOW structures. Values more than three standard deviations from the mean have been omitted, for clearer visualization. The top panel shows all predictions, where a small fraction of structures are predicted as nonmetals (in blue), the bottom panel shows only band gap predictions of structures classified as nonmetals.

Finally, we apply the best band gap regression model, i.e., the PaiNN ensemble to the full set of AFLOW structures. The distribution of its predictions can be seen in Figure 13. A large portion of structures are predicted to be metals (Figure 13, top panel). There is a wide distribution of band gaps for materials classified as metals. This is due to our regression models being trained on materials classified as nonmetal. Therefore, when the classification is metal the user should assume a band gap of zero. This demonstrates the importance of classification into metals and nonmetals before band gap prediction for our models. Only then can the band gap value be predicted meaningfully (Figure 13 bottom panel). This is the case for our band gap predictions for the entire AFLOW data set which are accessible at Zenodo ref (55). These predicted values should serve to enhance the database by providing users with new information that has demonstrated a low error on our test set and comes with an uncertainty estimate.

## Summary and Conclusions

To conclude, we find that our NAS yields ensemble models that significantly outperform models from the literature in terms of band gap and formation-energy regression. We find that the reference model^17^ performs well for band gap classification, being superior to the PLMF model despite being trained on formation energies. As expected, the materials with a calculated band gap slightly above zero are the most difficult to classify.

For band gaps, the best individual NAS models for both search spaces we explore (PaiNN and MPEU based) do not improve over the reference model on the test split. Our analysis shows that the latter performs significantly better on the test split as compared to the validation split, while our best NAS model yields similar results for both splits. Ensembling NAS candidates, however, does much better than any single model (reference model included). We find the NAS ensemble performs best when selecting the ten best models on the validation split. The ensemble of the NAS candidates outperforms different ensembling methods that use only the reference model in terms of test RMSE. This shows the benefit of the NAS. Our band-gap predictions using the best performing model for the entire AFLOW data set (more than 3.4 million data points) are accessible at Zenodo ref (55) and can be used for further investigations.

Analyzing the uncertainty of the MPEU NAS models, we find that the absolute errors of the ensemble models to be mostly below 50 and 10 meV for band gap and formation-energy regression, respectively, thereby approaching the numerical precision of DFT results. For band gap regression, we observe a significant correlation (of 0.63) in the uncertainty estimate for the ensemble. This also applies for data points including a Hubbard-*U* correction. The corresponding model is more certain and also less error-prone, while for formation energies, the trend is the opposite. We find that the ensemble model performs well for cubic structures but less well for triclinic materials since there are fewer training samples. In general, the best individual NAS model and ensemble identify the trends in formation energies and band gaps for polymorphs correctly. Predictions for oxides turned out to be anomalous when performing band gap classification despite their relative abundance in the data set. More work is required to explain this trend. Our findings may help to better understand when to apply such models and to motivate researchers to create balanced data sets with respect to structures and compositions.

This work demonstrates the usefulness of more complicated GNN architectures. Despite the relatively small size of the training data set, roughly 62,000 data points, the MPEU model that uses edge updates outperforms the SchNet model. The improved performance of the PaiNN ensemble over the MPEU model suggests that a more complex model, e.g., one that includes directional information, also results in better predictions of scalar properties, such as the band gap.

To further improve the NAS, one could use more complex search algorithms that are more exploitative (e.g., genetic or Bayesian optimization). This could, however, degrade the performance of the ensemble NAS model, since they will likely return less diverse top-ranked architectures. We also observe significant differences in the RMSE scores for the test and validation splits, which suggests that the MAE may be a better selection criterion for future work on NAS, since the MAE scores are better correlated between splits. The choice of test and validation split could also be improved by employing a more sophisticated splitting method, like twinning,^56^ which aims to find a more representative test or validation split.

Possible future applications of our work include material discovery by exploring much larger data spaces. The NAS and ensemble methods applied to MPEU, PaiNN, or similar models may also be used to explore more intricate material characteristics such as elastic, thermal, or transport properties. Also, the uncertainty that the model provides, may be used in an active-learning framework. Overall, our findings may motivate other researchers to employ this methodology and our code in very different applications, even beyond materials science.

## Supporting Information Available

Box plots of additional hyperparameters; model performance on validation split; scatter plot of MAE versus RMSE; histogram of formation-energy errors; additional violin- and box plots of errors depending on species and Hubbard correction (PDF)
