# Supplementary material for: Band-Gap Regression with Architecture-Optimized Message-Passing Neural Networks
Source: Chem Mater. 2025 Feb 12;37(4):1358–69. doi: 10.1021/acs.chemmater.4c01988 (PMC11867039; doi:10.1021/acs.chemmater.4c01988)
Supplement: Supplementary file 1 — cm4c01988_si_001.pdf [file cm4c01988_si_001.pdf]

Band-gap regression with architecture-optimized message-passing neural networks: Supporting information

Tim Bechtel,[*,†,‡] Daniel T. Speckhard,[†,‡] Jonathan Godwin,[¶,†] and Claudia Draxl[†,‡]

†*Humboldt-Universität zu Berlin, Zum Großen Windkanal 2, 12489 Berlin, Germany*

‡*Max Planck Institute for Solid State Research, Heisenbergstraße 1, 70569 Stuttgart, Germany*

¶*Orbital Materials, Oak House, Tanshire Park, Shackleford Road, Elstead, Surrey, United Kingdom, GU8 6LB*

E-mail: tim.bechtel@physik.hu-berlin.de

The additional plots shown here serve to better understand the model performance. We can see in Fig. 1 how different architecture parameters, not shown in the main text, affect the band gap model metrics. The NAS results for the tasks of learning formation-energies with MPEU, as well as band gaps and formation energies with PaiNN with respect to architecture and numerical parameters are depicted in Figs. 2, 3, and 4, respectively. Figs. 5 and 6 show that MAE and RMSE are well correlated with each other. During our NAS training we made the assumption that we can train our models to minimize the RMSE and evaluate them on the MAE. This plot proves our assumption to be correct, i.e., that the two variables are positively correlated. Minimizing the RMSE during training is easier than the MAE, since the absolute value has a discontinuous derivative. Fig. 7 shows the distribution of

the absolute errors in the formation energy in the AFLOW dataset. A scatter plot of the prediction standard deviation, obtained from the PaiNN ensemble and Monte-Carlo dropout MPEU is shown in Figs. 9 and 10, respectively as a function the prediction error, along with distribution of the prediction standard deviations. The distribution of the MC dropout uncertainty estimates are shown for the best NAS band gap MPEU regressor in Fig. 11. The distribution of the absolute errors in the formation-energy models as a function of the crystal structure can be seen in Fig. 8. The standard deviations from the best NAS model trained on formation energies are analyzed using Monte-Carlo Dropout. The result can be seen in Fig. 12.

The ensemble NAS model's performance on different materials as a function of the material class is shown in Fig. 13 for the formation-energy task and in Fig. 14 for band-gap regression. In both figures, we see that despite oxides being the majority class of materials in our dataset, they are not the best performing class in our dataset.

Application of the ensemble PaiNN model for formation energies on the full set of AFLOW structures is shown in Fig. 15, in the form of a parity plot.

Table 1: Regression metrics for different models, trained on AFLOW, showing their performance on the validation and test splits.

**Property**	**Model**	**RMSE**		**MAE**	
		Validation	Test	Validation	Test
E_g [meV]	Ensemble	434	379	183	168
	Best in NAS	468	469	208	205
	Reference	506	399	209	180
E_f [meV/atom]	Ensemble	62.5	56.3	15.7	15.0
	Best in NAS	65.4	65.4	21.7	21.0
	Reference	75.0	57.5	19.1	17.9

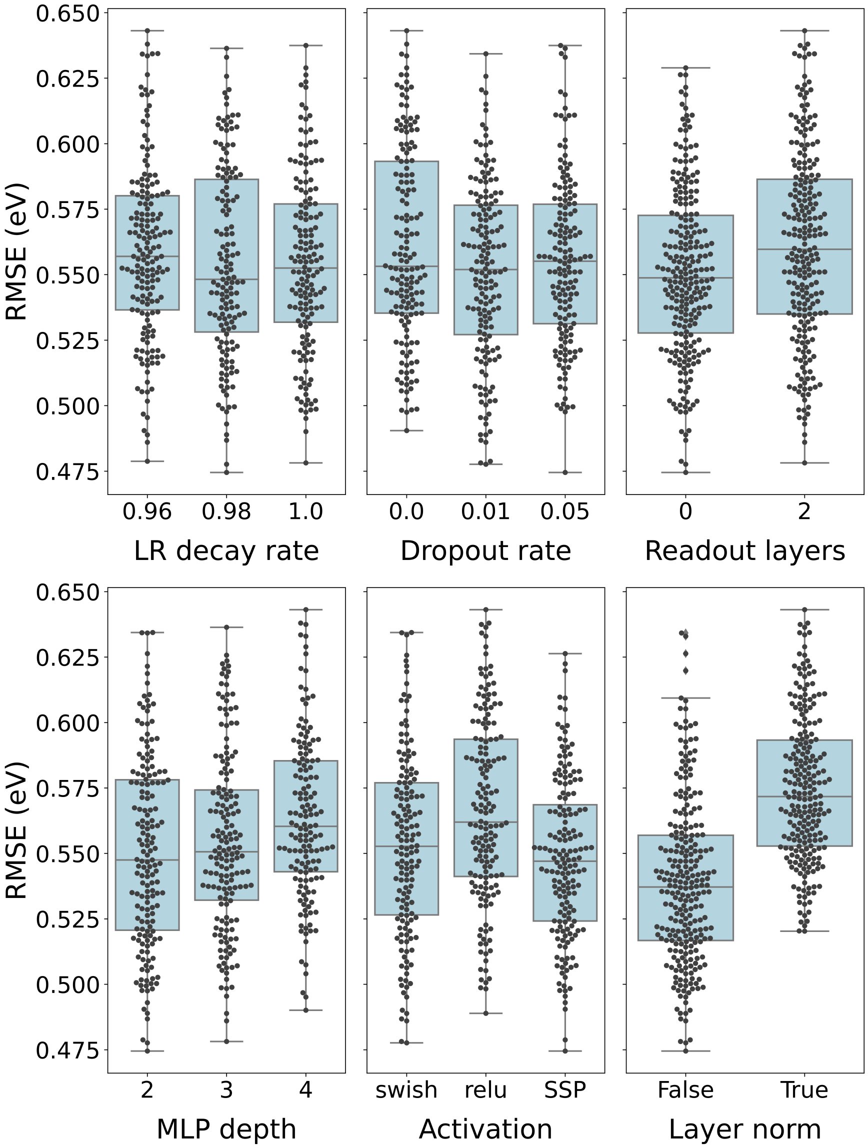

Figure 1: RMSE of the band-gap regression task for additional hyperparameters of the architecture search.

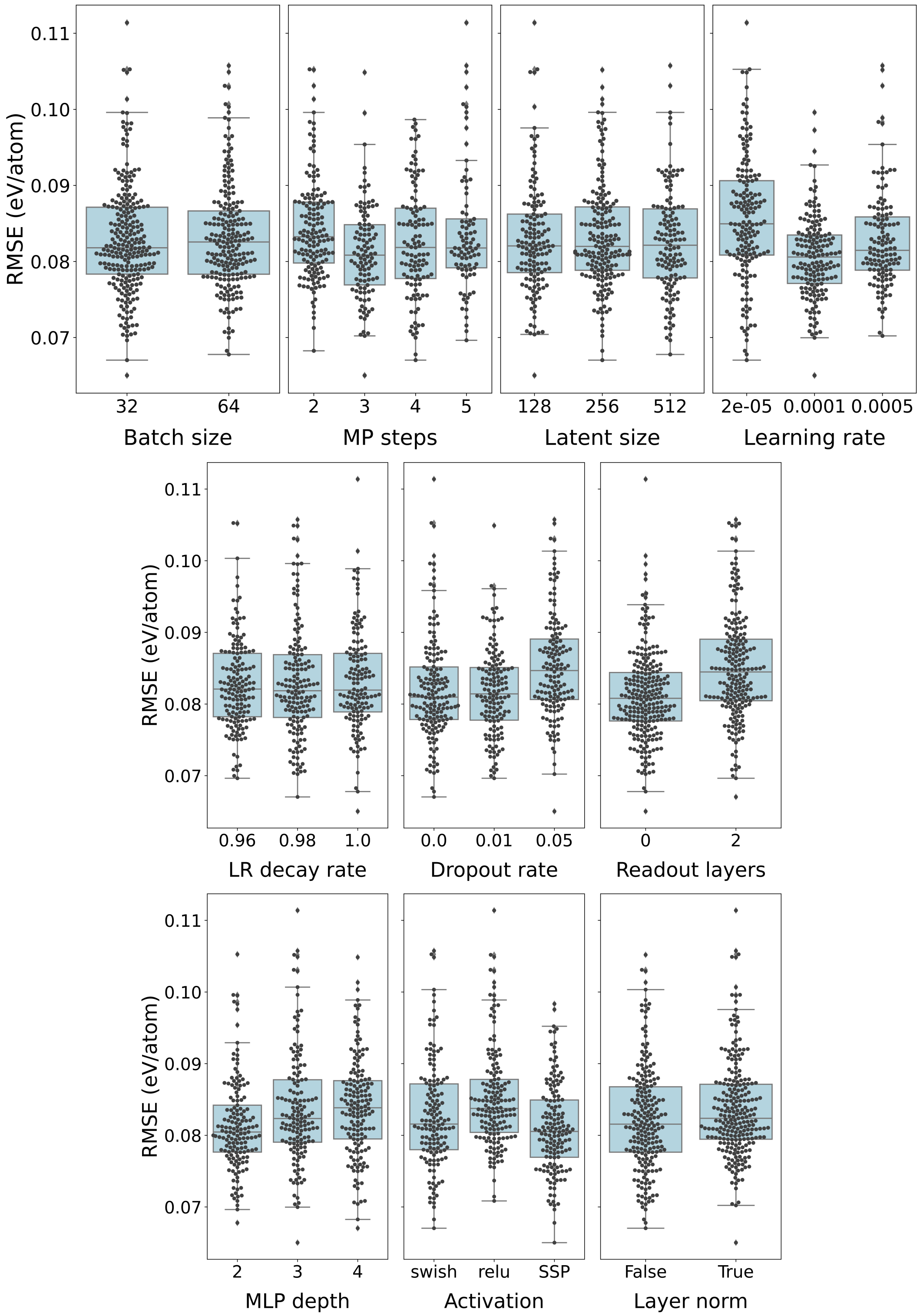

Figure 2: Result of the neural-architecture search for the best model that targets formation energies (in eV/atom) of AFLOW materials. The RMSE on the validation split is shown for several parameters and settings.

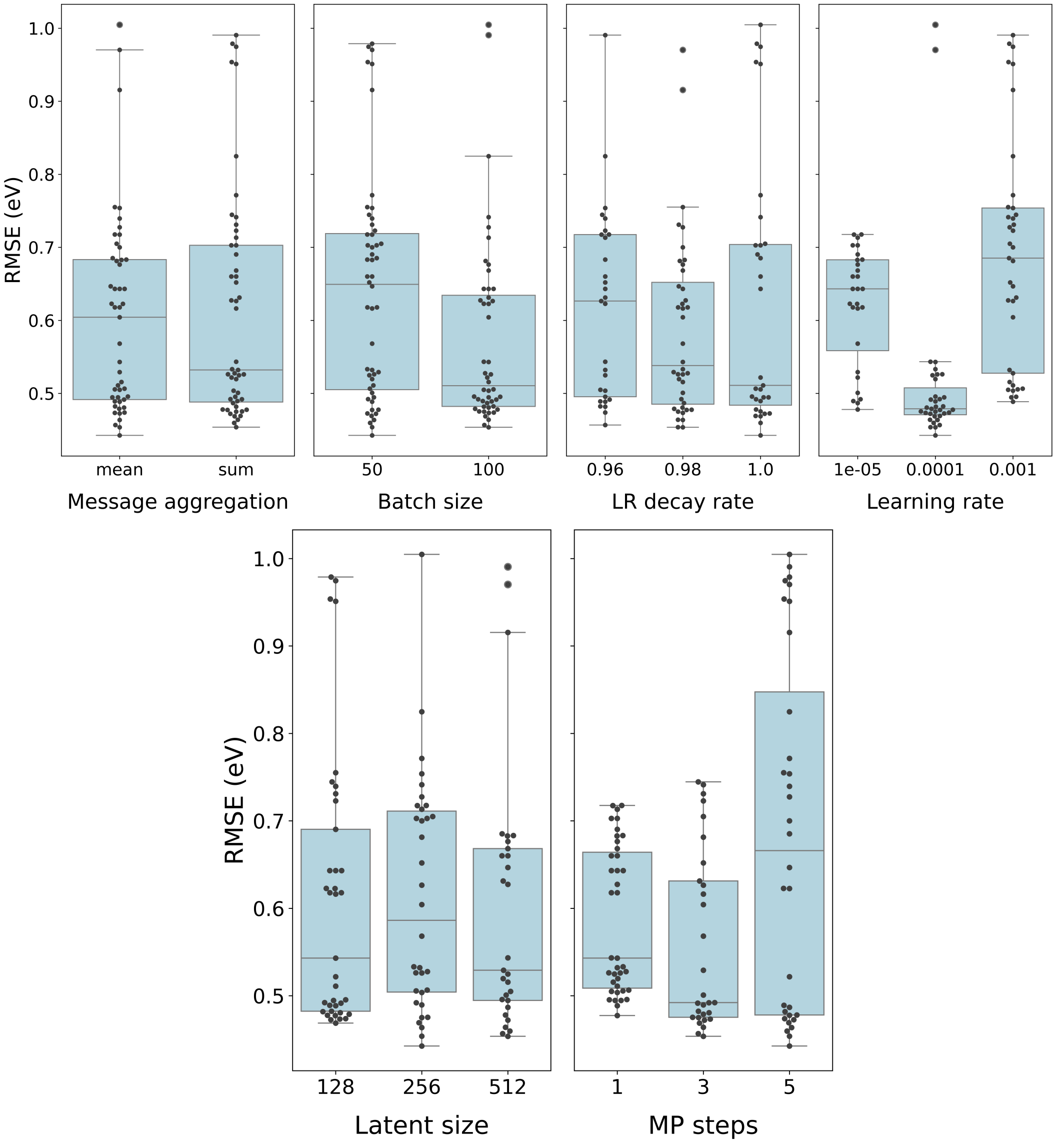

Figure 3: Result of the neural-architecture search for the PaiNN model on the band-gap regression task. The RMSE on the validation split is shown for several parameters and settings.

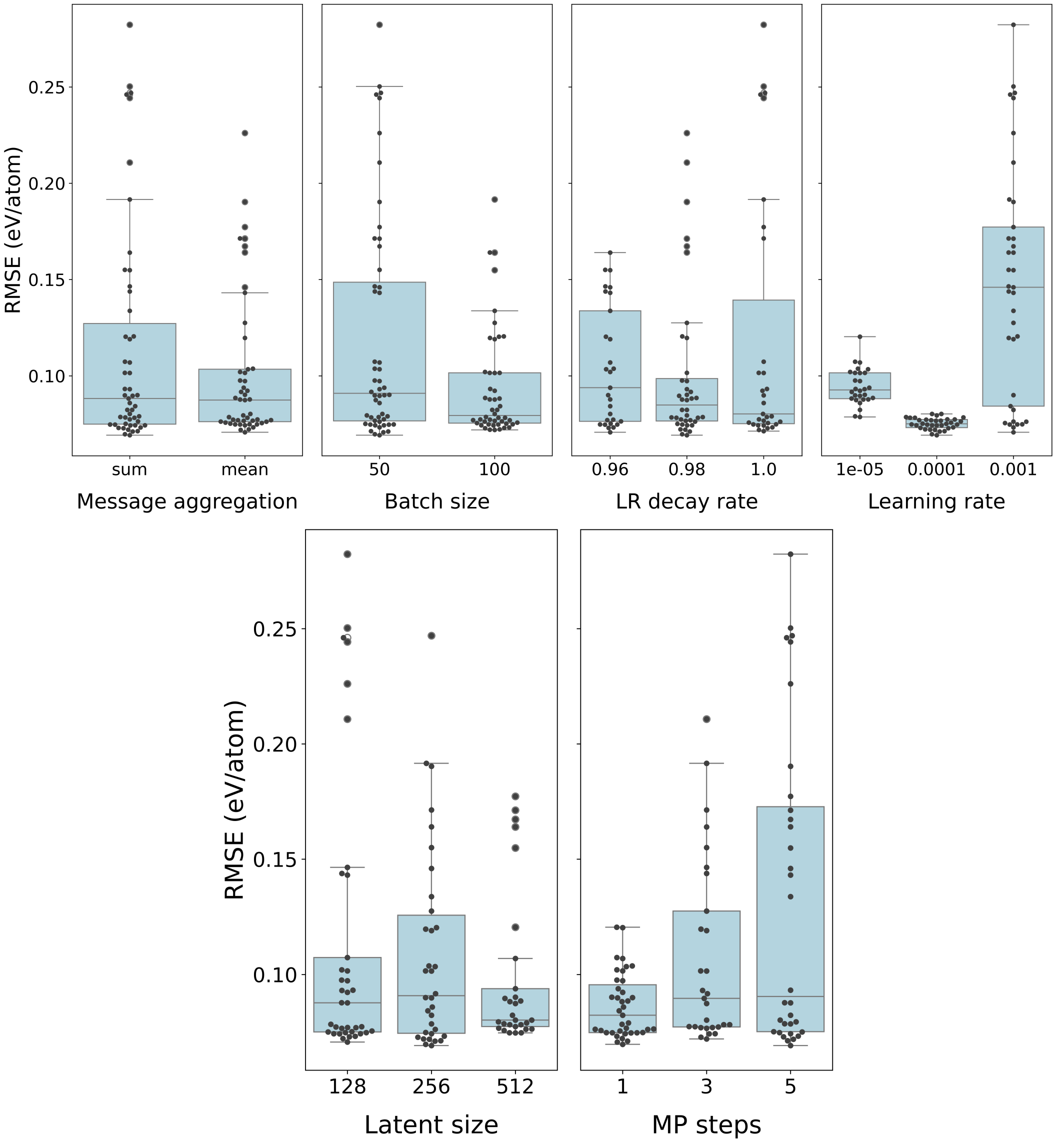

Figure 4: Result of the neural-architecture search for the PaiNN model on the formation-energy-regression task. The RMSE on the validation split is shown for several parameters and settings.

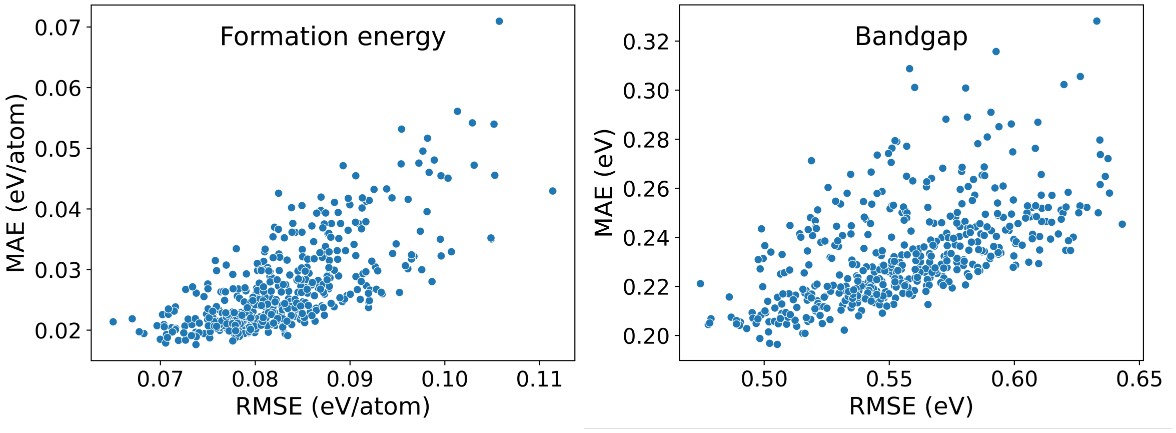

Figure 5: Distribution of mean-absolute errors (MAE) as a function of the RMSE for models in MPEU the architecture search.

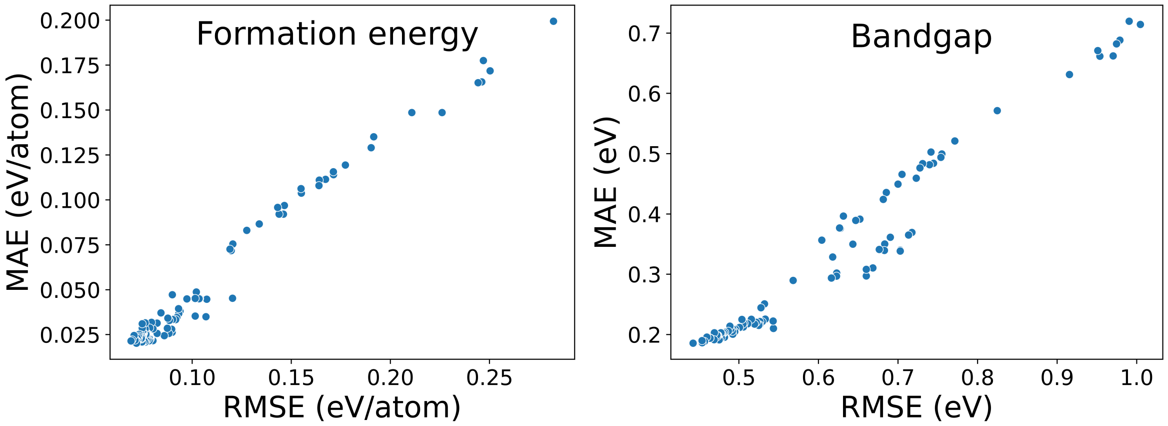

Figure 6: Distribution of mean-absolute errors (MAE) as a function of the RMSE for models in PaiNN the architecture search.

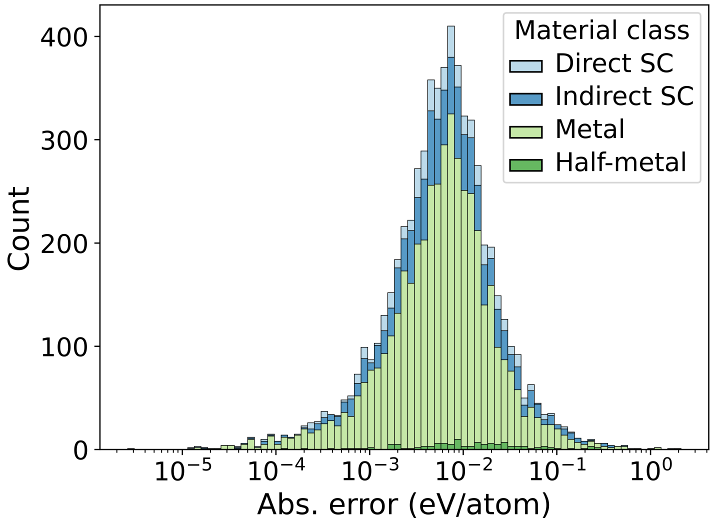

Figure 7: Distribution of absolute errors for different material classes in predicting formation energies for the AFLOW dataset, using the MPEU ensemble.

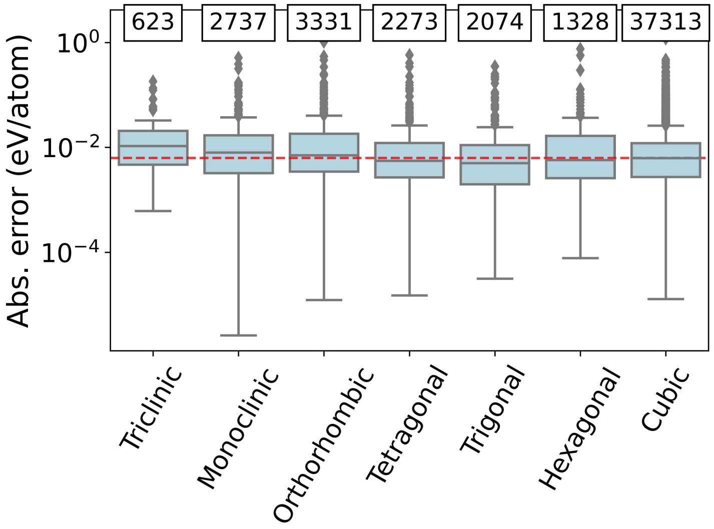

Figure 8: Distribution of absolute errors for different crystal systems in predicting formation energies for the AFLOW dataset, using the MPEU ensemble. The number of training structures for each crystal system is displayed at the top. The dashed red line shows the overall median error.

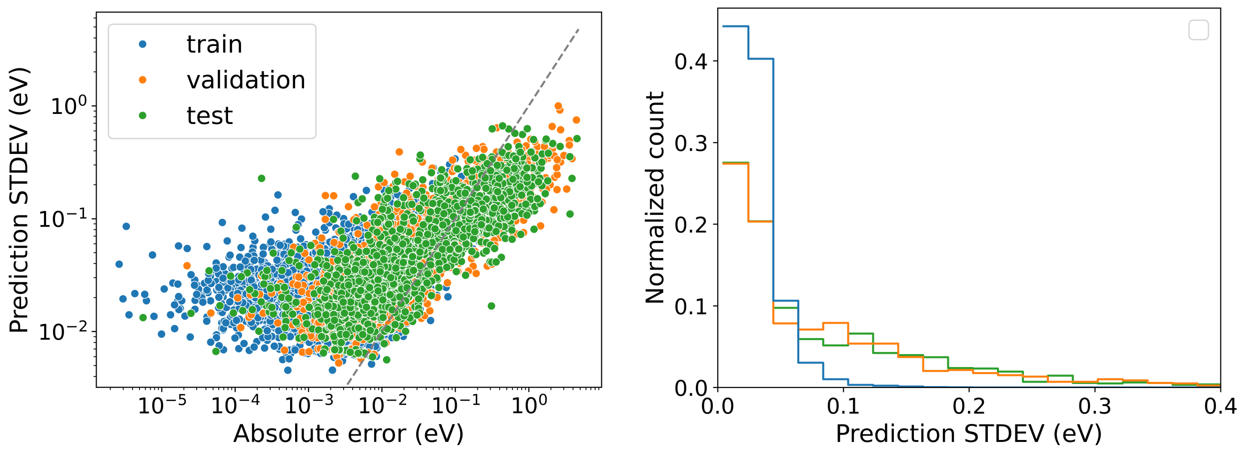

Figure 9: Scatter plot of ensemble band gap prediction variance depending on absolute ensemble prediction error (left), and histogram of prediction variances (right), for the three different data splits. Training and validation split points are partly hidden behind test split. Dashed line (left panel) shows perfect correlation for reference.

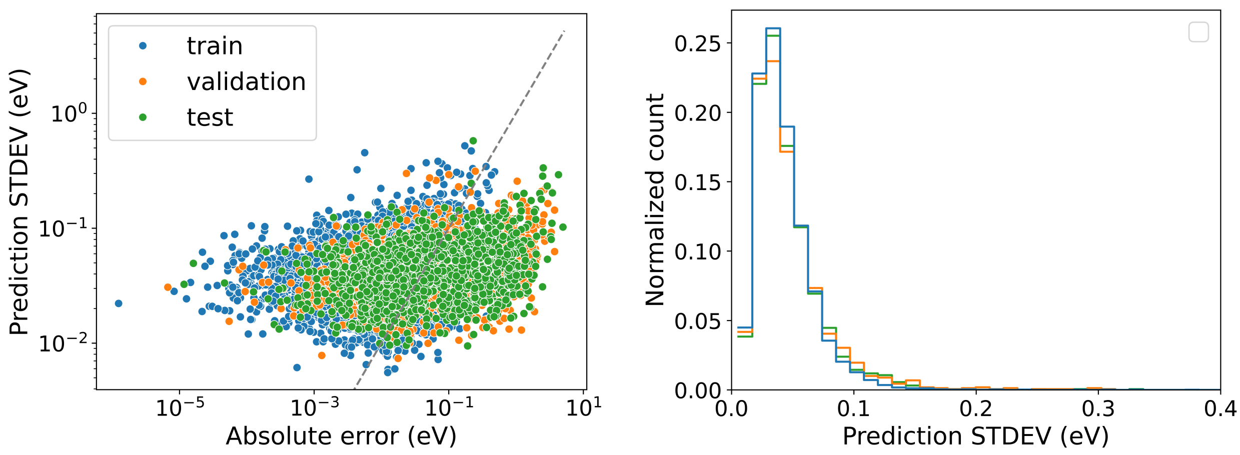

Figure 10: Scatter plot of Monte-Carlo dropout band gap prediction variance depending on absolute ensemble prediction error (left), and histogram of prediction variances (right), for the three different data splits. Training and validation split points are partly hidden behind test split. Dashed line (left panel) shows perfect correlation for reference.

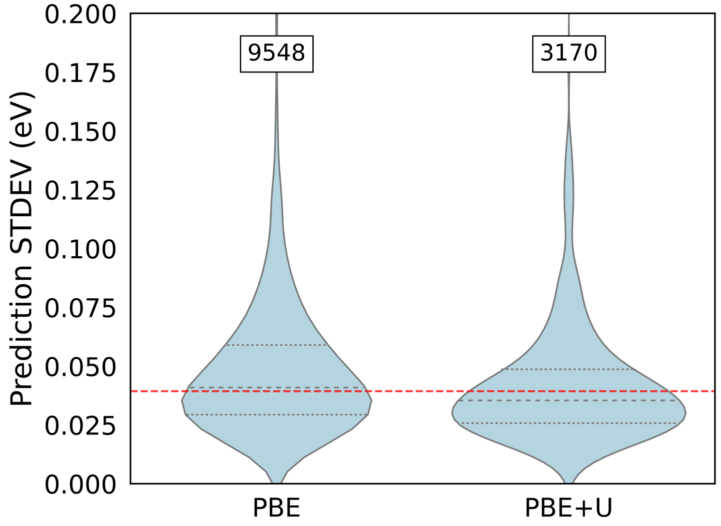

Figure 11: Violin plots of the standard deviations obtained by Monte-Carlo Dropout when predicting band gaps of identified non-metals, obtained by either PBE or PBE+U. The red horizontal line shows the median standard deviation over the whole test split, dashed lines show quartiles. The numbers of training examples are shown at the top.

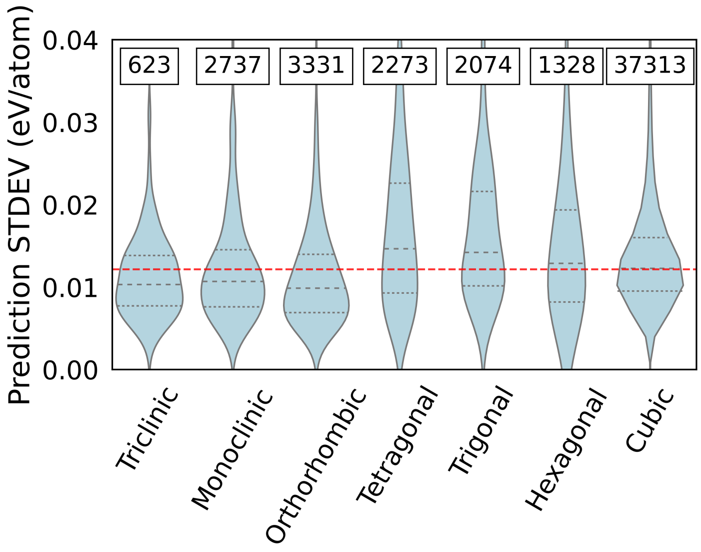

Figure 12: Violin plot of the standard deviations obtained by the Monte-Carlo Dropout when making formation-energy inferences. The numbers on top indicate the numbers of materials in the training split exhibiting the respective symmetry.

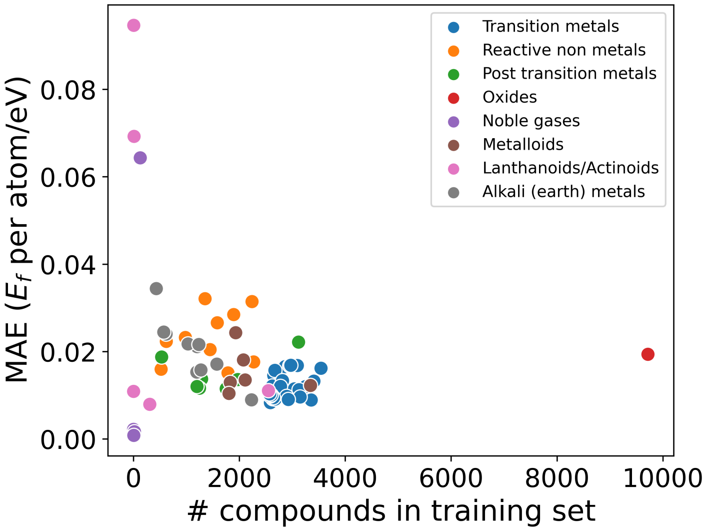

Figure 13: Mean absolute errors when predicting energy of formation, depending on number of materials in each material class present in the training split.

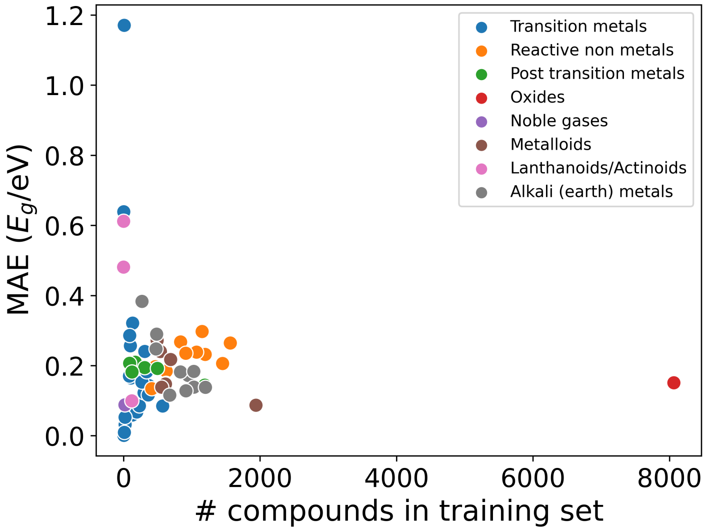

Figure 14: Mean absolute errors when predicting band gaps, depending on the number of materials with different species in the training split.

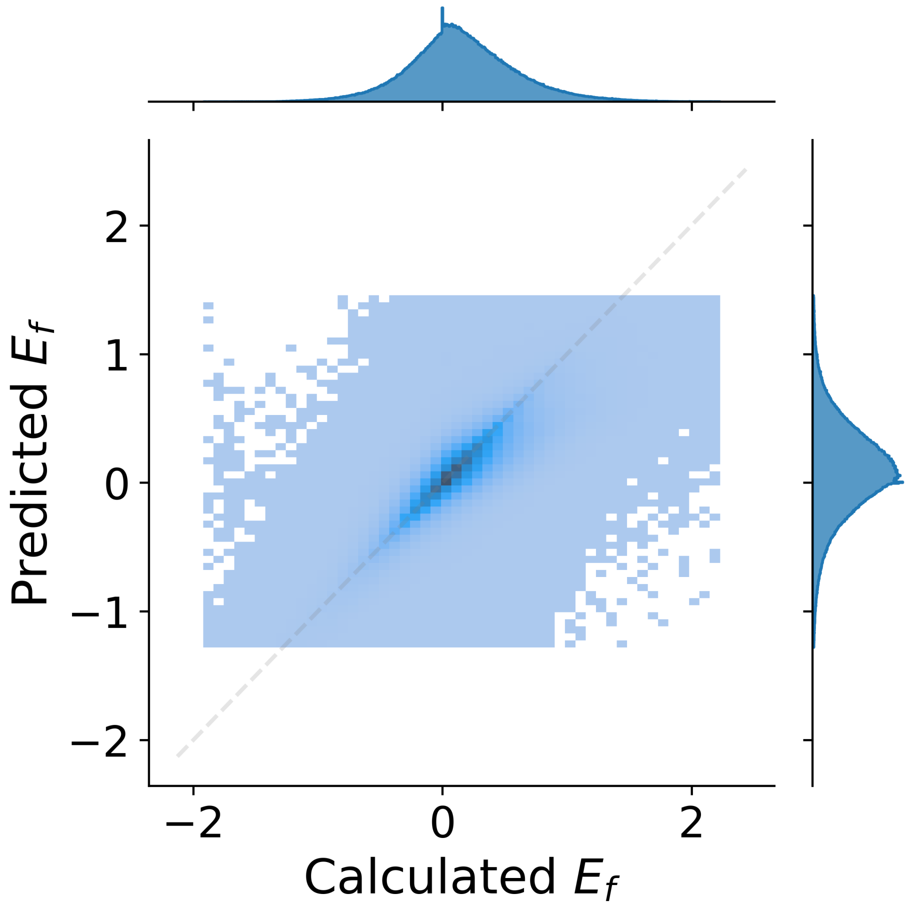

Figure 15: Parity histogram of the PaiNN ensemble applied to the full set of AFLOW structures. Values more than three standard deviations from the mean have been omitted on both axes, for clearer visualization. The dashed grey line on the diagonal shows where ideal predictions would lie.
